# Biosensor-Based Optimization of Cutinase Secretion by *Corynebacterium glutamicum*

**DOI:** 10.3389/fmicb.2021.750150

**Published:** 2021-10-28

**Authors:** Patrick J. Bakkes, Patrick Lenz, Carolin Müller, Astrid Bida, Doris Dohmen-Olma, Andreas Knapp, Marco Oldiges, Karl-Erich Jaeger, Roland Freudl

**Affiliations:** ^1^IBG-1: Biotechnology, Institute of Bio- and Geosciences, Forschungszentrum Jülich GmbH, Jülich, Germany; ^2^Institute of Molecular Enzyme Technology, Heinrich Heine University Düsseldorf, Forschungszentrum Jülich, Jülich, Germany; ^3^Institute of Biotechnology, RWTH Aachen University, Aachen, Germany

**Keywords:** *Corynebacterium glutamicum*, protein secretion, Sec-dependent export, signal peptide, fluorescence-based biosensor, EYFP, FACS, split GFP

## Abstract

The industrial microbe *Corynebacterium glutamicum* is gaining substantial importance as a platform host for recombinant protein secretion. We recently developed a fluorescence-based (eYFP) *C. glutamicum* reporter strain for the quantification of Sec-dependent protein secretion by monitoring the secretion-related stress response and now demonstrate its applicability in optimizing the secretion of the heterologous enzyme cutinase from *Fusarium solani pisi*. To drive secretion, either the poor-performing Pel^SP^ or the potent NprE^SP^ Sec signal peptide from *Bacillus subtilis* was used. To enable easy detection and quantification of the secreted cutinase we implemented the split green fluorescent protein (GFP) assay, which relies on the GFP11-tag fused to the C-terminus of the cutinase, which can complement a truncated GFP thereby reconstituting its fluorescence. The reporter strain was transformed with different mutant libraries created by error-prone PCR, which covered the region of the signal peptide and the N-terminus of the cutinase. Fluorescence-activated cell sorting (FACS) was performed to isolate cells that show increased fluorescence in response to increased protein secretion stress. Five Pel^SP^ variants were identified that showed a 4- to 6-fold increase in the amount and activity of the secreted cutinase (up to 4,100 U/L), whereas two improved NprE^SP^ variants were identified that showed a ∼35% increase in secretion, achieving ∼5,500 U/L. Most of the isolated variants carried mutations in the h-region of the signal peptide that increased its overall hydrophobicity. Using site-directed mutagenesis it was shown that the combined mutations F11I and P16S within the hydrophobic core of the Pel^SP^ are sufficient to boost cutinase secretion in batch cultivations to the same level as achieved by the NprE^SP^. Screening of a Pel^SP^ mutant library in addition resulted in the identification of a cutinase variant with an increased specific activity, which was attributed to the mutation A85V located within the substrate-binding region. Taken together the biosensor-based optimization approach resulted in a substantial improvement of cutinase secretion by *C. glutamicum*, and therefore represents a valuable tool that can be applied to any secretory protein of interest.

## Introduction

*Corynebacterium glutamicum* is a well-established microbial host for industrial biotechnology that is also gaining importance as a potent platform organism for the secretory production of recombinant proteins (Lee et al., 2016; Liu et al., 2016; Freudl, 2017). Secretion of recombinant proteins into the culture medium can be a preferred strategy as it possesses several key benefits over intracellular protein production (Quax, 1997). Targeted secretion can (i) avoid the intracellular accumulation of target proteins susceptible to aggregation (e.g., inclusion body formation), (ii) bypass endogenous toxic cellular effects and (iii) evade degradation by intracellular proteases. The diderm gram-positive bacterium *C. glutamicum* is a particularly attractive secretion host, as it secretes only few innate proteins into the culture medium and lacks significant extracellular proteolytic activity (Vertès, 2013). Moreover, several interesting target proteins such as therapeutic antibodies or lipases require the formation of intramolecular disulfide bonds for complete folding, which is usually restricted in the reducing environment of the cytoplasm (Saaranen and Ruddock, 2013; Freudl, 2017). The export of such proteins into the non-reducing extra-cytoplasmic milieu thus may promote the formation of biologically active protein. Finally, the secretory production of recombinant proteins obviates the need for cell disruption, thus enabling simple protein recovery and purification, which in turn, facilitates downstream processing and reduces the overall production costs (Quax, 1997). The mechanism(s) and protein systems involved in protein translocation across the mycolic acids of the outer membrane of *C. glutamicum* are as of yet unknown.

In bacteria, the vast majority of extra-cytoplasmic proteins are exported across the cytoplasmic membrane by the general secretion (Sec) pathway (Freudl, 2017; Prabudiansyah and Driessen, 2017; Rapoport et al., 2017). Characteristically, the Sec substrate proteins carry an N-terminal signal peptide, which is crucial for targeting to the Sec translocase (Rusch and Kendall, 2007). In addition to their role in protein targeting and translocation, signal peptides can also influence the biosynthesis, the folding kinetics and the stability of the dedicated substrate protein (Deana et al., 1998; Freudl, 2018). Despite a general lack of sequence homology, Sec signal peptides share a universally conserved tripartite organization comprising a positively charged N-terminal region, a central hydrophobic core (h-region), and a polar C-terminal domain (c-region) that contains the cleavage site recognized by the signal peptidase (Izard and Kendall, 1994; Freudl, 2018). All three regions are known to effectively contribute to the export of Sec substrates (Izard and Kendall, 1994; Freudl, 2018).

The Sec system has been exploited for the secretion of a wide variety of recombinant proteins in a range of different microorganisms (Choi and Lee, 2004; Freudl, 2017; Cui et al., 2018; Hamed et al., 2018; Neef et al., 2021). However, finding a suitable signal peptide capable of driving efficient secretion remains a major bottleneck, mainly because it is still not possible to reliably predict the secretion performance of an individual signal peptide in context with the desired heterologous target protein and the applied expression host (Freudl, 2018). Hence, extensive screenings of a large variety of signal peptides seems to be the most-promising approach to identify the best-performing signal peptide. Signal peptide diversity can be achieved by the creation of multiple variants of a specific signal peptide using mutagenesis approaches (Caspers et al., 2010) or by generation of libraries from a large number of different microbial signal peptides (Brockmeier et al., 2006; Watanabe et al., 2009; Degering et al., 2010; Hemmerich et al., 2016). Besides the use of large signal peptide libraries for heterologous protein secretion in *C. glutamicum* there are many studies, too numerous to be listed in their entirety here, that report on the use of single (or a small number of different) signal peptides for the efficient secretion of a variety of different target proteins. Comprehensive overviews of the used signal peptides and the obtained amounts of the secreted target proteins, which include amongst others α-amylase, (endo)xylanase, Phospholipase C, cutinase and M18 scFv are given in Lee et al. (2016) Liu et al. (2016), and Freudl (2017).

In addition to the identification of a suitable signal peptide for secretion, efforts to improve the yield of a secreted target protein usually requires the optimization of a multitude of (co-dependent) biological and bioprocess parameters (Hemmerich et al., 2019). The monitoring of protein secretion during such optimization process however is frequently limited by the availability of an easy functional assay for the desired target protein. In addition, the multitude of potential optimization parameters requires the testing of a high number of samples, which necessitates a general assay for the monitoring of protein secretion compatible with high-throughput approaches.

**FIGURE 1:**
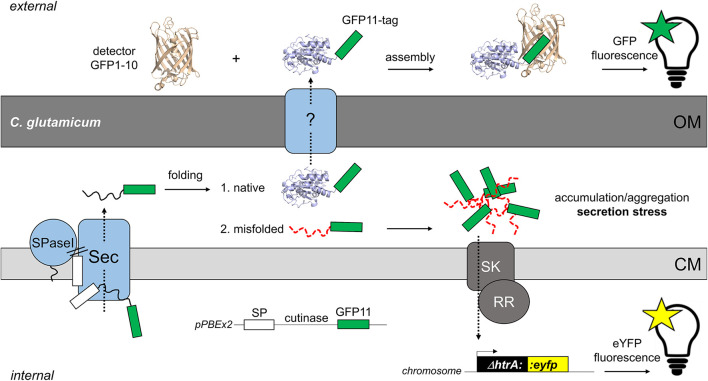
Fluorescence-based monitoring of Sec-secretion stress in *C. glutamicum* and detection of secreted target protein by the split GFP assay. The overproduction of secretory proteins promotes accumulation of incompletely or misfolded proteins at the membrane-cell envelope interface. To alleviate this secretion stress, cells typically respond by the upregulation of the extra-cytoplasmic protease HtrA, which is able to degrade the misfolded proteins at the *trans*-side of the cytoplasmic membrane (CM). In *C. glutamicum*, a two-component system consisting of a sensory kinase (SK) and a response regulator (RR) is likely involved in regulation of expression of the adjacent *htrA* gene (Jurischka et al., 2020). In the *C. glutamicum* K9 biosensor strain, the *htrA* gene has been replaced by the *eyfp* gene, while leaving the *htrA* signal transduction pathway intact. The fluorescent reporter strain allows the monitoring of Sec-dependent secretion of recombinant proteins in a dose-dependent manner (Jurischka et al., 2020). The actual mechanism(s) and protein systems facilitating the transport of proteins across the mycolic acid outer membrane (OM) of *C. glutamicum* still remain an enigma. For detection of the target protein in the extracellular medium, we adapted the split GFP technology (Cabantous et al., 2005; Cabantous and Waldo, 2006) that previously had been adopted to monitor recombinant protein secretion *in B. subtilis* (Knapp et al., 2017). For this purpose, the eleventh β-sheet of GFP, is fused via a flexible linker to the C-terminus of the target protein (in our studies the model enzyme cutinase). The non-fluorescent detector GFP, which lacks eleventh β-sheet (GFP1-10) is produced separately in *E. coli* and purified from inclusion bodies. Combining the GFP11-tagged protein with the detector GFP1-10 enables reconstitution of functional fluorescent GFP. Subsequent measurement of the split GFP fluorescence allows detection and quantification of the secreted target protein. The white box indicates the signal peptide (SP); the green box indicates the GFP11-tag; SPase, signal peptidase.

For such purposes, we recently developed a fluorescent *C. glutamicum* reporter strain that is capable of quantifying Sec-dependent export of recombinant proteins by monitoring the related secretion stress, independent of an assay for the respective target protein (Jurischka et al., 2020). The mechanistic principle of this protein secretion biosensor is drawn schematically in Figure 1. High-level production of secretory proteins in *C. glutamicum* induces a stress response, which is triggered by the accumulation of incompletely or incorrectly folded secretory protein molecules at the cytoplasmic membrane-cell envelope interface (Kleine et al., 2017; Jurischka et al., 2020). Bacteria typically respond to such secretion stress by the upregulation of the housekeeping protease HtrA (Raivio and Silhavy, 2001; Meltzer et al., 2009; Kleine et al., 2017; Jurischka et al., 2020), which is capable of degrading the misfolded proteins at the *trans*-side of the cytoplasmic membrane. Replacement of the chromosomal *htrA* gene in *C. glutamicum* ATCC13032 (Kinoshita et al., 1957) by the gene coding for the yellow fluorescent protein eYFP, while leaving the *htrA* signal transduction pathway intact, resulted in a reporter strain that allows the monitoring of Sec-dependent export of recombinant proteins by means of its fluorescence output. The fluorescence of the reporter strain was shown to respond to the secretion of different recombinant proteins in a dose-dependent manner and allowed the differentiation between the performances of distinct signal peptides that were used to drive the secretion of cutinase from *Fusarium solani pisi* (Jurischka et al., 2020). Moreover, high-level secretory production of cutinase in the reporter strain resulted in highly fluorescent cells that can be separated from poorly secreting, low fluorescent cells using a fluorescence-activated cell sorter (Jurischka et al., 2020).

In the present study, we further investigated the applicability of the fluorescent biosensor strain in optimizing the secretion of the cutinase. Considering the fact that (large) libraries of signal peptides from different Sec proteins are not easily accessible to everyone and that reports on comparative analyses of signal peptides are rather scarce, the selection of a single signal peptide for secretion based on its performance described in literature can be a more useful approach. However, such arbitrarily selected signal peptide might not be optimal for the desired target protein and applied expression host, and thus may require optimization. Hence, a suitable alternative to testing many signal peptides of different origin is the improvement of a single signal peptide by mutagenesis approaches. We created different libraries by random mutagenesis of genes encoding signal peptide-cutinase fusions that were expressed in the reporter strain and screened by FACS to identify cells that exhibited improved cutinase secretion. For efficient Sec-dependent protein export not only the signal peptide is crucial (Izard and Kendall, 1994; Freudl, 2018), also amino acids downstream the signal peptide (N-terminus of the mature protein or “export initiation domain”) have been shown to contribute to the export efficiency (Andersson and von Heijne, 1991; Musik et al., 2019). Therefore, mutagenesis was performed on the coding region for the signal peptide and a substantial part of the N-terminal domain of the mature cutinase.

To facilitate detection and quantification of the secreted cutinase, we applied the split green fluorescent protein (GFP) technology (Cabantous et al., 2005; Cabantous and Waldo, 2006) that was previously successfully adapted to monitor heterologous protein secretion in an activity-independent manner in *B. subtilis* (Knapp et al., 2017). Here, the eleventh β-sheet of GFP (GFP11) was fused to the C-terminus of the cutinase. This short GFP11-tag is able to complement a truncated, non-fluorescent GFP1-10 protein added to the culture medium after cultivation, thereby enabling reconstitution of the GFP fluorescence, which can be used as read-out for the amount of secreted cutinase (Figure 1). The results of our present study clearly show that secretion of the heterologous model protein cutinase by *C. glutamicum* can be substantially improved by employing our Sec secretion biosensor as a tool for the identification of superior signal peptide variants, bypassing the need for a direct assay for the optimization of the secretion of a desired target protein.

## Materials and Methods

### General Procedures

The bacterial strains and plasmids used in this study are shown in Supplementary Table S1. For standard cultivations, *E. coli* was grown in LB medium (Bertani, 1951) containing 50 μg/mL kanamycin at 37°C, while *C. glutamicum* was grown in BHIS medium containing 37 g/L brain heart infusion (BHI, Difco Laboratories), 91 g/L sorbitol and 25 μg/mL kanamycin at 30°C. Standard protocols were followed for DNA manipulations (Green and Sambrook, 2012). FastDigest restriction enzymes, T4 DNA ligase, FastAP thermosensitive Alkaline Phosphatase and Phusion DNA Polymerase were supplied by Thermo Fisher Scientific (Langerwehe, Germany) and used according to the manufacturer’s specifications. Oligonucleotides were obtained from Eurofins (Ebersberg, Germany). For DNA amplification, appropriate PCR conditions were set for each primer pair, and PCR products were purified using the NucleoSpin gel and PCR clean up kit from Macherey-Nagel (Düren, Germany). Recombinant plasmids were isolated from *E. coli* or *C. glutamicum* using the Nucleospin plasmid purification, or NucleoSnap Plasmid Midi kit from Macherey-Nagel (Düren, Germany). DNA sequencing was carried out by Eurofins (Ebersberg, Germany). DNA concentrations were determined using a NanoDrop spectrophotometer (Thermo Fisher Scientific). Protein concentrations were determined by a BCA assay (Thermo Fisher Scientific), using BSA as a protein standard.

### Plasmid Constructions

Initial plasmid constructions were carried out using the established *E. coli*/*C. glutamicum* shuttle vector pEKEx2 (Eikmanns et al., 1994). In a recent study however, we uncovered that the substantial leakiness of the *tac* promoter from pEKEx2 is caused by a reduced function of a modified plasmid-encoded *lac* repressor, while replicate DNA sequences in the pEKEx2 backbone contribute to plasmid instability (Bakkes et al., 2020). For tightly controlled secretory production of recombinant proteins, we therefore turned to the improved expression vector pPBEx2, which is a cured derivative of pEKEx2 (Supplementary Table S1; Bakkes et al., 2020). The plasmids, oligonucleotides and general procedures used to create the various expression constructs are shown in the Supplementary Material accompanying this paper.

### Construction of Different Mutant Libraries

For the construction of a library of mutants of the cutinase-GFP11 fused to the Pel signal peptide (Pel^L1^), the DNA region encompassing the Pel signal peptide (Pel^SP^), linker and partial cutinase gene up to the internal *Kpn*I site was amplified from pPBEx2-Pel-cutinase-GFP11. For this purpose, the GeneMorph II Random Mutagenesis Kit (Agilent Technology, Santa Clara, CA, United States) and the oligonucleotide pair p07 and p08 were used (Supplementary Table S2). Conditions for error-prone PCR (epPCR) were set, such that approximately 3 - 5 mutations were introduced per PCR fragment. Subsequently, the epPCR products were purified, cleaved with *Pst*I and *Kpn*I, in conjunction with *Dpn*I, which eliminates the template DNA, and then ligated with gel-purified pPBEx2-Pel-cutinase-GFP11 that had been cleaved with *Pst*I and *Kpn*I. Typically, multiple ligation-transformation reactions were performed. For each ligation reaction (20 μL), 50 ng of cut vector and a 5 to 7-fold molar excess of cut insert was used. Ligation reactions were pooled and then purified. Next, multiple transformation reactions were carried out, for each using 50 μL of electrocompetent *E. coli* One Shot TOP10 cells (Supplementary Table S1) and 2 μL of the purified ligation mixture. Electroporation was carried out using a Gene Pulser Xcell (BioRad) and 0.1 mm electroporation cuvettes, with device settings: 1800 V, 25 μF and 200 Ω. Each batch of transformed cells was spread on a large square LB-agar plate containing 50 μg/mL kanamycin. To assess the library size, different amounts of a single batch of transformed cells were spread out. The Pel^L1^ library size was ∼1.8 ⋅ 10^5^. All plates were incubated overnight at 37°C. The colonies were then suspended and pooled in a volume of 100 mL LB medium containing 50 μg/mL kanamycin and grown for 5 h at 37°C and 120 rpm, after which the cells were used for plasmid isolation. Next, multiple transformation reactions were carried out, using 1.3 – 2 μg of library DNA per 100 μL of electrocompetent cells of the biosensor strain *C. glutamicum* K9 (Supplementary Table S1; Jurischka et al., 2020). Electroporation was performed essentially as described earlier (Eggeling and Bott, 2005). The transformation reactions were spread on large square BHIS-agar plates containing 15 μg/mL kanamycin and then incubated for two days at 30°C. Hereafter, the colonies were suspended and pooled in a total volume of 100 mL BHIS medium containing 25 μg/mL kanamycin and grown for 5 h at 30°C and 120 rpm. Finally, the Pel^L1^ cells were supplemented with glycerol to a final concentration of 30% (v/v), flash-frozen using liquid nitrogen and then stored at –80°C, until use. To create the second-generation library Pel^L2^, epPCR mutagenesis was performed on plasmid from variant V3 (P16S), which was isolated from the Pel^L1^ mutant library after FACS-based screening. For creation of the mutant library based on the NprE signal peptide (NprE^L^), plasmid pPBEx2-NprE-cutinase-GFP11 was used as DNA template in epPCR reactions. For both Pel^L2^ and NprE^L^, primers p07 and p08 (Supplementary Tables S1, S2) were used for DNA amplification. *E. coli* One Shot TOP10 electrocompetent cells were transformed with the mutant libraries Pel^L2^ and NprE^L^ and then treated in the same manner as described for Pel^L1^. Estimated library sizes were ∼5.8 ⋅ 10^5^ and ∼2.4 ⋅ 10^5^, respectively.

### Site-Directed Mutagenesis

Site-directed mutagenesis (SDM) was performed using the Q5 Site-Directed Mutagenesis Kit from New England BioLabs (Frankfurt am Main, Germany) according to the manufacturer’s recommendations. The primers that were used to introduce the desired point mutations in the DNA region coding for the Pel^SP^ or the cutinase are listed in Supplementary Table S2 (p09 – p18). For amplification of the nearly 9 kb plasmid fragments, 2% (v/v) DMSO was included in the reaction mixture and an extension time of 60 s per 1 kb was used. The various template and primer combinations used to create the different variants are shown in Supplementary Table S3. Subsequently, kinase, ligase and *Dpn*I treatment were performed according to the manufacturer’s instructions. Finally, 2 μL of the reactions mixtures were used to transform chemically competent *E. coli* One Shot TOP10 cells. Recombinant plasmids were then isolated from single clones, verified by DNA sequencing and subsequently electroporated into the *C glutamicum* K9 biosensor strain as described by Eggeling and Bott (2005).

### Standard Cultivation of *Corynebacterium glutamicum* K9 Biosensor Cells for Cutinase Secretion Experiments

For fluorescence-based monitoring of cutinase secretion, the *C. glutamicum* K9 biosensor cells carrying the different recombinant plasmids (Supplementary Table S1) were grown in BHIS medium in a 48-well flowerplate (m2p-labs, Baesweiler, Germany) in a BioLector (m2p-labs, Baesweiler, Germany) at 1,200 rpm, 85% relative humidity and 30°C for 6 h. Next, 50 μL of the BHIS precultures were transferred to a new flowerplate containing 800 μL CGXII minimal medium (Keilhauer et al., 1993) and 1% (v/v) glucose in each well and growth was continued overnight in the BioLector. Hereafter, the optical density (OD_600_) of the cultures was determined and appropriate amounts were used to inoculate fresh CGXII minimal medium containing 1% (v/v) glucose in a new flowerplate, yielding a starting OD_600_ of ∼1 and cultivation was continued in the BioLector. Four hours after inoculation, IPTG was added to a final concentration of 250 μM and cultivation was sustained overnight. During cultivation, flowerplates were covered with a sterile gas permeable membrane to avoid evaporation. Bacterial growth was monitored online by measuring the backscatter at 620 nm (gain 20). For measuring online fluorescence of the biosensor cells, the BioLector is equipped with an eYFP filter module (λ_ex_ 508 nm, λ_em_ 532 nm). The eYFP biosensor fluorescence was measured using a signal gain of 90. The specific fluorescence of the biosensor cells was calculated by dividing the eYFP fluorescence (AU) by the corresponding backscatter (AU). The output of the biosensor cells was plotted as the specific fluorescence over time after subtraction of the corresponding specific fluorescence of the empty vector control cells (Δspecific fluorescence). The graphs thus depict the net difference in fluorescence response triggered by the secretory production of the cutinase. At the end of each cultivation, cell-free culture supernatants were prepared by centrifugation at 10,000 × *g* for 5 min and the levels of extracellular cutinase were assessed by SDS-PAGE analysis and/or complementary split GFP fluorescence and cutinase activity measurements.

### Fed-Batch Cultivation of *Corynebacterium glutamicum* K9 Biosensor Cells for Cutinase Secretion

*Corynebacterium glutamicum* K9 biosensor cells carrying the recombinant plasmids as indicated were grown in 50 mL CGXII minimal medium containing 20 g/L glucose and 30 μg/ml kanamycin at 30°C and 300 rpm (25 mm shaking diameter). Cells were harvested in the late exponential phase and suspended in 0.9% (w/v) NaCl. The fed-batch cultivation was performed in a MTP-MF32-BOH1 flowerplate covered with F-GPRSMF32-1 sealing foil in a BioLector Pro (m2p-labs, Baesweiler, Germany), allowing for microfluidic pH control and fed-batch operation. Cultivation conditions were 30°C, 1,400 rpm, ≥30% headspace oxygen and ≥85% relative humidity. Biomass and pH were measured using a cycle time of 13 min. Per well, 800 μl CGXII minimal medium containing 22 mg/L protocatechuic acid, 30 μg/mL kanamycin and 5 g/L glucose (while urea was omitted) were inoculated with the corresponding preculture cells to an OD_600_ of 0.5. After 10 h, 400 g/L glucose were fed with 0.16 μL pump volume and a constant feed rate of 5.22 μL/h. After one hour, the pH in all wells was controlled one-sided to a set point of 6.8 with 3 M KOH, medium PI setting and 0.3 μL pump volume. Cutinase gene expression was induced with 250 μM IPTG after 8 h and cells were harvested after ∼25 h. The supernatants at the end of the cultivation were analyzed for cutinase content and activity.

### Split Green Fluorescent Protein Assay

To determine the amounts of secreted cutinase-GFP11 protein, split GFP fluorescence measurements were conducted off-line at the end of the cultivations. The split GFP assay was carried out essentially as described previously (Knapp et al., 2017). In brief, the non-fluorescent detector GFP1-10 was produced in *E. coli* BL21(DE3) cells carrying pET22b-sfGFP1-10 and then purified from the inclusion bodies fraction. For reconstitution of holo-GFP, 20 μL of the *C. glutamicum* culture supernatants containing the GFP11-tagged cutinase were mixed with 180 μL of detector GFP1-10 solution in each well of a black flat-bottom 96-well microtiter plate (mtp). Culture supernatants of cells harboring the empty vector pPBEx2 served as controls. The mtp was then covered with a sterile gas permeable membrane to avoid evaporation and then incubated for 24 h at 20°C under gentle agitation to allow assembly of functional GFP. Finally, split GFP fluorescence was measured using an Infinite M1000 Pro plate reader (Tecan, Crailsheim, Germany). Excitation was performed at 485 nm (bandwidth 10 nm), while the fluorescence emission was recorded from 505 to 550 nm in 5 nm steps and using an appropriate gain factor (typically in the range of 105 – 120). The GFP-specific emission maximum at 510 nm was used for calculations. For each recombinant strain, at least two independent clones were cultivated and measurements were performed at least in duplicate.

### Cutinase Activity Assays

Cutinase activity in culture supernatants was determined spectrophotometrically using *p*-nitrophenyl palmitate (*p*NPP) as a substrate analogon, as described previously (Bakkes et al., 2020). For each recombinant strain, at least two independent clones were tested and measurements were always performed in duplicate. In addition, cutinase production was assessed by an *in situ* activity assay on agar plates. For this purpose, 3 μL of BHIS overnight cultures of *C. glutamicum* K9 biosensor cells carrying the different recombinant plasmids were spotted on BHIS-agar plates containing 400 μM IPTG, 1% (v/v), Tween-20 (substrate for cutinase) and 25 μg/mL kanamycin. The plates were incubated for at least 2 days at 30°C. The formation of optically turbid zones is indicative of production of enzymatically active cutinase (Bakkes et al., 2020).

### SDS-PAGE Analysis

12.5% SDS-PAGE analysis was carried out essentially as described previously (Bakkes et al., 2020). In brief, at the end of the cultivation, the OD_600_ was determined and the cells were separated from the CGXII culture medium by centrifugation. Proteins from 500 μL culture supernatant were precipitated using a final concentration of 10% (v/v) trichloroacetic acid. Precipitated proteins were dissolved in pH-adjusted SDS-PAGE sample buffer (Laemmli, 1970). The amount of secreted protein was normalized to the cell density at the end of the cultivation and volumetric amounts corresponding to 1.5 OD_600_ cell equivalents were applied to each lane.

### Flow Cytometry

Single-cell fluorescence measurements and cell sorting experiments were performed on a BD FACSAria Fusion cell sorter (BD Biosciences, Franklin Lakes, NJ, United States), using a 70 μm nozzle and a sheath pressure of 70 psi. A 488 nm blue solid laser was used for excitation. The forward-scatter characteristics (FSC) were recorded as small-angle scatter, whereas the side-scatter characteristics (SSC) were recorded as orthogonal scatter of the 488 nm laser. For measurements of the eYFP fluorescence of the *C. glutamicum* K9 biosensor cells, a 502 nm long-pass and 530/30 nm band-pass filter set was used. In all cases, prior to data acquisition, debris and electronic noise were excluded from the analysis by electronic gating in a SSC-H *vs*. FSC-H scatter plot. From the resulting population (P1) doublets were next excluded by a second gating step in a FSC-W *vs*. FSC-H scatter plot. The resulting population (P2) was then always used for fluorescence acquisition. Prior to FACS analysis/sorting, biosensor cells were appropriately diluted (typically OD_600_ < 0.1) in CGXII rudimentary solution pH 7.0, containing 20 g/L (NH_4_)_2_SO_4_, 5 g/L urea, 1 g/L KH_2_PO_4_, 1 g/L K_2_HPO_4_, 0.25 g/L MgSO_4_⋅7H_2_O, and 42 g/L MOPS. Cell samples were then passed through a 50 μm filter to remove any particulate matter and then subjected to FACS analysis. Cells were sorted into 5 mL polystyrene collection tubes (Sarstedt, Nürnbrecht, Germany) loaded with 2 mL CGXII medium containing 1% (v/v) glucose, 25 μg/mL kanamycin and appropriate amount of IPTG (typically 250 μM). For liquid sorting, precision was always set to purity, with the event rate never exceeding 16,000 events per second. Sorted cell samples were then split in half and transferred to a flowerplate for enrichment cultivation in a BioLector as described above. The cells were grown until the stationary phase was reached (24–48 h) and then subjected to FACS analysis and single cell sorting. Single cells were sorted onto OmniTrays^TM^ (Thermo Fisher Scientific) containing BHIS-agar and 15 μg/mL kanamycin, using the single-cell sort precision mode along with an event rate of ∼6,000 – 8,000 s^–1^. The agar plates were incubated for at least 2 days at 30°C to allow colony development. FACSDiva 8.0.2 (BD Biosciences, San Jose, United States) was used for FACS control and data analysis. A graphical workflow of the FACS analysis and sorting experiments is depicted in Supplementary Figure S1.

## Results and Discussion

### Monitoring Cutinase Secretion Using the *Corynebacterium glutamicum* K9 Biosensor Strain in Combination With the Split Green Fluorescent Protein Assay

To demonstrate the applicability of the protein secretion biosensor strain, we aimed at improving the cutinase secretion driven by signal peptides showing a different secretion performance. For this purpose, the signal peptides from the *B. subtilis* enzymes pectate lyase (Pel^SP^) and neutral protease (NprE^SP^) were selected. The Pel^SP^ is known to facilitate high-level cutinase secretion in *B. subtilis* (Brockmeier et al., 2006), whereas only low levels are achieved in *C. glutamicum* (Hemmerich et al., 2016). The NprE^SP^ on the other hand conveys cutinase secretion by *B. subtilis* at only low level (Brockmeier et al., 2006), whereas it is one of the best performing signal peptides for cutinase secretion in *C. glutamicum* (Hemmerich et al., 2019; Jurischka et al., 2020).

**FIGURE 2:**
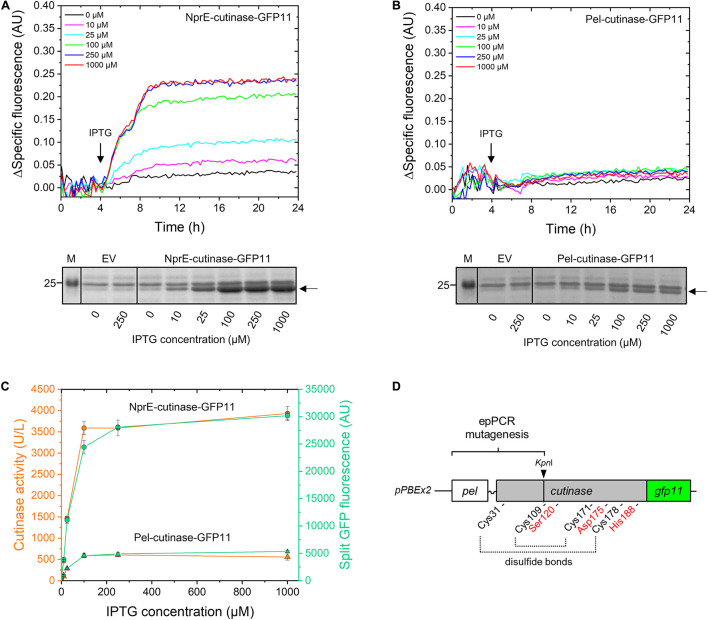
Monitoring the secretion of GFP11-tagged cutinase using the *C. glutamicum* K9 biosensor strain and the split GFP assay. *C. glutamicum* K9 biosensor cells harboring pPBEx2 (EV), pPBEx2-NprE-cutinase-GFP11 or pPBEx2-Pel-cutinase-GFP11 were grown in CGXII medium containing 1% (v/v) glucose in a BioLector for 24 h at 30°C, 1,200 rpm and 85% relative humidity. Four hours after inoculation, IPTG was added to the cultures to indicated final concentrations. The fluorescence response of the biosensor cells during cultivation is shown in the upper panels as the Δspecific fluorescence over time for cells expressing NprE-cutinase-GFP11 **(A)** or Pel-cutinase-GFP11 **(B)**. Cutinase-GFP11 in the respective culture supernatants after 24 h of growth was analyzed by SDS-PAGE (lower panels). The proteins were visualized by Coomassie Brilliant Blue staining. EV; *C. glutamicum* K9 biosensor cells harboring pPBEx2 (empty vector). The arrows indicate the position of the processed (signal peptide-less) cutinase-GFP11 (expected size 25.1 kDa). M, marker proteins with Mw indicated in kDa. **(C)** In parallel, culture supernatants were analyzed for cutinase activity and split GFP fluorescence. **(D)** Schematic view of the epPCR mutagenesis range, which spans the Pel^SP^, the linker region and a substantial portion of the mature N-terminal part of the cutinase gene up to the unique internal *Kpn*I restriction site. Positions of amino acids important for cutinase activity are indicated; i.e., the catalytic triad Ser120-Asp175-His188 and the four cysteines involved in disulfide bond formation.

First, the performance of Pel^SP^ and NprE^SP^ in facilitating the secretion of the GFP11-tagged cutinase was investigated (Figure 2). For this purpose, *C. glutamicum* K9 biosensor cells carrying pPBEx2-NprE-cutinase-GFP11 (Figure 2A) or pPBEx2-Pel-cutinase-GFP11 (Figure 2B) were induced with different concentrations of IPTG. K9 biosensor cells carrying the empty vector (EV) served as control. The fluorescence response of the cells to protein secretion stress over time in dependence of the IPTG concentration is shown in Figures 2A,B (upper panels). The secretion performance was assessed by determining the levels of the extracellular cutinase-GFP11 at the end of the cultivation by means of SDS-PAGE (Figures 2A,B, lower panels) and by measuring the split GFP fluorescence and the cutinase activity in the culture supernatant (Figure 2C).

The secretory production of NprE-cutinase-GFP11 leads to substantial secretion stress as evidenced by the eYFP fluorescence response of the biosensor cells upon induction with IPTG (Figure 2A, upper panel). The amount of the specific fluorescence at the end of the cultivation of the respective biosensor cells correlated with the amounts of the extracellular cutinase-GFP11 as determined by SDS-PAGE, showing a dose-dependent response (Figure 2A, lower panel). A similar dose-dependency was observed when NprE-cutinase without a GFP11-tag was secreted (*cf.* Supplementary Figure S2). However, in this case, the specific fluorescence values at the end of the cultivation were slightly lower (∼0.03–0.05 AU), which may indicate that the GFP11-tag marginally contributes to increased secretion stress. The secretory production of Pel-cutinase-GFP11 on the other hand did not induce an obvious dose-dependent biosensor response (Figure 2B). In fact, even at saturating IPTG concentration (1 mM) the specific fluorescence at the end of the cultivation was only slightly higher than that of cells that were not exposed to IPTG. In accordance with the low fluorescence output of the biosensor cells, only low amounts of cutinase-GFP11 were present in the culture supernatants, which confirms the weak secretion performance of the Pel^SP^ (Figure 2B, lower panel).

In parallel, the amounts of extracellular cutinase-GFP11 were quantitatively assessed by measuring the split GFP fluorescence (Figure 2C). Importantly, for both Pel^SP^ and NprE^SP^ a good correlation exists between the results from the split GFP assay, the activity assay and the amounts of the secreted cutinase-GFP11 as visualized by SDS-PAGE (*cf.* Figures 2A–C). Thus, the split GFP assay is capable of quantification of the extracellular cutinase, which is underlined by a linear correlation between the applied amount of cutinase-GFP11 and the resultant split GFP fluorescence (Supplementary Figure S3). These results are in excellent agreement with cutinase-GFP11 secretion studies performed in *B. subtilis* (Knapp et al., 2017). In this respect, it is important to note that although the GFP11-tag marginally contributed to secretion stress, the tag did not have a noticeable negative effect on the amount and activity of the secreted cutinase (Supplementary Figure S2). However, for cutinase-GFP11 secreted by *B. subtilis* a small inhibitory effect on the lipolytic activity was noted (Knapp et al., 2017). These observations may indicate a more efficient folding of the cutinase-GFP11 in the host *C. glutamicum* after Sec-dependent export across the cytoplasmic membrane. Thus, the split GFP technology was successfully adapted to monitor recombinant protein secretion by *C. glutamicum*.

### Construction and Fluorescence-Activated Cell Sorting-Based Screening of Pel^SP^-Cutinase-GFP11 Mutant Libraries

In a first step, we aimed at improving the cutinase secretion driven by the Pel^SP^. To identify Pel-cutinase-GFP11 variants that exhibit improved secretion, first a library of Pel-cutinase-GFP11 mutants was generated via error-prone PCR. Mutagenesis was performed on the Pel^SP^ coding region and a sizeable part of the N-terminal domain of the mature cutinase comprising amino acids 2–88 (Figure 2D). The signal peptide is crucial for efficient Sec-dependent protein export (Izard and Kendall, 1994; Freudl, 2018) and thus an obvious target for mutagenesis. By mutagenizing also the N-terminal part of the mature cutinase, enzyme variants with improved secretion properties and/or activity may be generated. In this respect, it is important to note that the catalytic triad Ser120-Asp175-His188, and 3 out of 4 cysteines involved in disulfide bond formation, all of which are crucial for cutinase activity, were not subject to mutagenesis (Figure 2D).

The Pel-cutinase-GFP11 mutant library was cloned into pPBEx2 and subsequently used to transform *E. coli*. The plasmids were then isolated from approximately 1.8 ⋅ 10^5^ colonies and used to transform the *C. glutamicum* K9 biosensor strain. The resulting transformants (Pel^L1^) were pooled for further analysis. The Pel^L1^ cells were cultivated along with the control strains Pel^SP^, NprE^SP^ and empty vector (EV) in CGXII medium containing 1% v/v glucose in a BioLector at 30°C and 1,200 rpm at 85% relative humidity. Four hours after inoculation, IPTG (250 μM final concentration) was added to the cultures to induce the secretory production of cutinase-GFP11 and growth was continued overnight. The overnight cultures were then appropriately diluted for FACS analysis. For all FACS experiments, a preselection of cells was performed to exclude cell doublets and cell debris by gating in a dot plot of FSC-H against FSC-W (Table 1, gate P2). The resultant gate P2 typically contained ∼93% of the total number of cells (1.0 ⋅ 10^5^) analyzed (Table 1).

**TABLE 1:** Fluorescence-activated cell sorting analysis of *C. glutamicum* K9 biosensor cells carrying the Pel^L1^ mutant library or control plasmids ^a,b^.

Biosensor cells carrying	Gate P2		Gate P3-1	
	Events	Median fluorescence (AU)	Events (% of P2)	Median fluorescence (AU)
Empty vector (EV)	93,149 ± 194	443 ± 13	139 ± 12 (0.1%)	994 ± 17
NprE-cutinase-GFP11 (NprE^SP^)	92,683 ± 237	624 ± 10	2,326 ± 136 (2.5%)	1,140 ± 7
Pel-cutinase-GFP11 (Pel^SP^)	92,965 ± 80	477 ± 4	186 ± 18 (0.2%)	1,001 ± 6
Mutant library (Pel^L1^)	93,184 ± 353	521 ± 4	1,128 ± 93 (1.2%)	1,366 ± 11
Pel^L1^ after enrichment	93,471	810	12,112 (13%)	1,327

*^a^A detailed graphical depiction of the workflow is shown in Supplementary Figure S1.*

*^*b*^1.0 ⋅ 10^5^ cells of each strain were analyzed. In all cases, electronic gating (gate P2) was performed in a dot plot of FSC-H against FSC-W to exclude cell doublets and cell debris. Selection of cells with high median fluorescence was performed by appropriately setting gate P3-1 in a dot plot (Supplementary Figure S4), respectively. The number of cells contained within the respective gates, as well as the median fluorescence (AU) of these cells are indicated.*

The biosensor cells expressing Pel-cutinase-GFP11 exhibited a median fluorescence of 477 AU, which was only ∼8% higher than that of the corresponding control cells carrying the empty vector. In contrast, biosensor cells secreting cutinase-GFP11 via the NprE^SP^ exhibited a median fluorescence that was 41% higher than that of the empty vector control cells (624 *vs*. 443 AU, respectively). These results are consistent with the notion that the low-level cutinase secretion facilitated by the Pel^SP^ imposes only minor stress on the cells, whereas the high-level secretion achieved by the NprE^SP^ causes substantial stress to the cells. In comparison to the wild-type (WT) Pel^SP^ cells, the Pel^L1^ cells exhibited a 9% increase in median fluorescence (i.e., 521 *vs.* 477 AU), which indicates an overall increase in secretion stress. The corresponding graphs showing the eYFP fluorescence plotted against the cell size (FSC-H) are shown in Supplementary Figure S4.

To select for Pel^L1^ variants potentially exhibiting improved secretion, a sort gate (P3-1) was set to include as many of the highly fluorescent cells as possible, thereby excluding the cells with lower fluorescence. i.e., the less productive cells (Table 1 and Supplementary Figure S4). Sort gate P3-1 contained on average 1128 Pel^L1^ cells, constituting the top 1.2% of the P2 population, while approximately six-fold fewer Pel^SP^ control cells (i.e., 186) fell into this gate (Table 1). The selected Pel^L1^ cells exhibited a median fluorescence of 1366 AU, which is 36% higher than that of the Pel^SP^ control cells.

Next, 2.0 ⋅ 10^5^ Pel^L1^ cells were sorted from gate P3-1 and collected together in CGXII medium containing 1% (v/v) glucose and 250 μM IPTG. The pooled cells were cultivated in a BioLector until the stationary phase was reached and then analyzed by FACS. Importantly, now more than 12,000 Pel^L1^ cells fell into gate P3-1, indicating successful enrichment (>10-fold) of highly fluorescent cells (median fluorescence of 1327 AU). In addition, a control sorting experiment was carried out by performing enrichment of Pel^L1^ cells that exhibited a near average fluorescence. For this purpose, a gate (P3-M) was set to encompass the center of the P2 population (median fluorescence of 486 AU), thereby excluding the highly fluorescent cells (Supplementary Figure S4). Next, 2.0 ⋅ 10^5^ Pel^L1^ cells were collected from gate P3-M and cultivated in a BioLector until the stationary phase was reached.

After the enrichment cultivation, individual cells were sorted from both the P3-1 culture and the P3-M control culture. For both cultures, the pre-set gate P3-1 was used to sort single cells onto BHIS-agar plates. After colony outgrowth, 88 clones each were transferred to liquid BHIS medium and cultivated overnight at 30°C and 900 rpm. To demonstrate feasibility of the sorting strategy, assessment of the cutinase secretion was carried out by performing an *in situ* activity assay. For this purpose, aliquots of the respective overnight cultures were spotted on Tween-agar plates containing IPTG to induce cutinase expression. Importantly, a substantially higher number of cutinase producing clones was obtained from the highly fluorescent cell population (P3-1) than from the control population (P3-M), i.e., ∼32% *vs.* ∼10%, respectively (Supplementary Table S4). The relatively low number of clones producing active cutinase present in both populations indicates that the introduction of mutations by error-prone PCR in most cases is detrimental to the secretory production and/or activity of the cutinase. When a more restrictive sort gate (P3-2) was applied, which typically collects the top 0.3% fluorescent cells (median fluorescence of ∼1800 AU) selectivity was increased to ∼43%. Thus, in all cases, sorting of the highly fluorescent Pel^L1^ cells resulted in an increased selectivity for clones producing enzymatically active cutinase, indicating a successful sorting strategy (Supplementary Table S4). A graphical workflow of the cultivation conditions and the FACS-based experiments is shown in Supplementary Figure S1.

### Analysis of the Secretion Performance of Variants Isolated From the Pel^SP^-Cutinase-GFP11 Mutant Libraries

**FIGURE 3:**
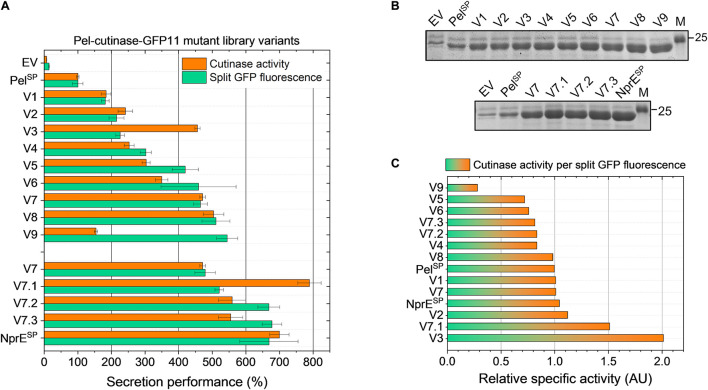
Secretion performance of the variants isolated from the Pel^SP^-cutinase-GFP11 mutant libraries. Plasmids were isolated from the clones isolated after FACS-based screening of the primary mutant library Pel^L1^ (V1–V9) and the second-generation library Pel^L2^ based on V7 (V7.1–V7.3) and then re-introduced in the *C. glutamicum* K9 biosensor strain. The recombinant strains were grown in CGXII medium containing 1% (v/v) glucose in a BioLector for 24 h at 30°C, 1,200 rpm and 85% relative humidity along with the control strains harboring pPBEx2 (EV), pPBEx2-NprE-cutinase-GFP11 (NprE^SP^) or pPBEx2-Pel-cutinase-GFP11 (Pel^SP^). Four hours after inoculation, IPTG was added (250 μM) to the cultures. At the end of the cultivation (24 h) the amount and activity of the extracellular cutinase-GFP11 was determined by **(A)** split GFP fluorescence and cutinase activity measurements, respectively. The secretion performance is shown relative to that of the wild-type Pel-cutinase-GFP11 (Pel^SP^), which was set to 100%. **(B)** Complementary SDS-PAGE analysis of the extracellular levels of cutinase-GFP11 (expected size 25.1 kDa). The proteins were visualized by Coomassie Brilliant Blue staining. M, marker proteins with Mw indicated in kDa. **(C)** The relative specific activity for each variant was obtained by dividing the cutinase activity (%) by the split GFP fluorescence (%). The specific activity of the indicated variants relative to Pel^SP^ is shown in ascending order.

The secretion performance of nine clones isolated by FACS from Pel^L1^ was analyzed in more detail. For this purpose, plasmid DNA was isolated from the original clones and then re-introduced in the K9 biosensor strain. The resulting recombinant strains were cultivated along with the control strains EV, Pel^SP^ and NprE^SP^ in a BioLector, using 250 μM IPTG to induce expression. The secretion performance of the different strains was assessed at the end of the cultivation (24 h) by determining the amount and activity of the extracellular cutinase (Figure 3).

Notably, in comparison to the control strain in which cutinase-GFP11 secretion is driven by the WT Pel^SP^ (set to 100%), all nine isolated Pel^L1^ variants (V1 – V9) showed a substantial improvement of the secretion performance (Figure 3). Overall, the different variants showed a 2- to nearly 6-fold increase in split GFP fluorescence, while extracellular cutinase activities were 2- to 5-fold increased (Figure 3A). The amounts of extracellular cutinase-GFP11 as determined by SDS-PAGE were consistent with the split GFP fluorescence measurements (*cf*. Figures 3A,B, upper panel).

DNA sequence analysis of the plasmids isolated from the respective strains revealed 25 DNA mutations, of which 17 resulted in an amino acid substitution (Table 2). As might be expected, all isolated variants carried mutations within in the signal peptide region. Interestingly, several variants showed mutations at identical positions in the signal peptide, i.e., Phe11 in V1, V3, V5, and V9, and Pro16 in V7 and V8 (Table 2). These residues may therefore represent potential hotspots for improving the Pel^SP^-driven cutinase secretion.

**TABLE 2:** Overview of the variants obtained after FACS-based screening of the Pel-cutinase-GFP11 mutant libraries (Pel^L1^ and Pel^L2^).

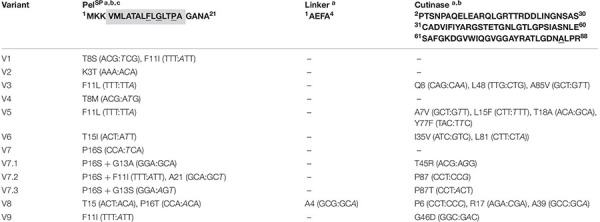

*^*a*^The DNA coding region that was subjected to random mutagenesis included the Pel^*SP*^, the small linker, and a sizeable part of the cutinase (amino acids 2–88); the corresponding amino acids of the respective parts of the wild-type fusion protein are indicated. The altered DNA triplets and the corresponding amino acid changes of the isolated variants are indicated.*

*^*b*^Key amino acids that were exchanged by site directed mutagenesis are indicated in bold and underlined.*

*^*c*^The putative h-region of the Pel^*SP*^ is indicated by a gray box.*

To investigate if secretion can be further improved, a second-generation mutant library (Pel^L2^) was constructed based on one of these hotspot variants. For this purpose, V7 plasmid was selected as the template DNA for error-prone PCR, since it contains only a single DNA mutation that leads to the P16S exchange in the Pel^SP^ (Table 2), which increases the secretion performance by more than fourfold (Figure 3A). Interestingly, V8, which shows a slightly better secretion performance, contains a similar amino acid exchange at the same position, i.e., P16T, while carrying an additional five silent mutations (Table 2). The screening of the Pel^L2^ mutant library was carried out in an identical manner as described above for Pel^L1^. Three novel variants (V7.1 – V7.3) were obtained that showed a substantial improvement of the secretion performance in comparison to the parental V7 (Figure 3). The levels of the secreted cutinase-GFP11 achieved by these variants were similar to those achieved by the initially far superior NprE^SP^ (Figures 3A,B, lower panel). DNA sequencing revealed that V7.1 and V7.3 both carry an additional amino acid substitution besides P16S at the identical position in the signal peptide (Table 2). The identified mutations G13A and G13S likely contribute to the improved cutinase secretion. In case of V7.2, the additional signal peptide mutation F11I was identified. The fact that 5 of the 12 clones isolated from the mutant libraries carry a F11I/L mutation (Table 2), strengthens the notion that these mutations have an important contribution to the increased secretion performance (Figure 3).

It is of note that many of the isolated variants in addition to the signal peptide mutations also carry mutations in the cutinase region (Table 2), which may affect the cutinase secretion and/or activity. However, most of the isolated variants exhibited a specific activity (cutinase activity per split GFP fluorescence) close to that of the Pel^SP^ control (Figure 3C). Noticeable exceptions are V3 and V7.1, which show a substantial increase in specific activity, and V9, which exhibits a substantial decrease in specific activity. DNA sequence analysis identified a single amino acid substitution in the cutinase of each of these variants; A85V in V3, T45R in V7.1 and G46D in V9 (Table 2). It is reasonable to assume that these mutations are primarily responsible for the observed effects on the enzyme activity.

### Impact of the Different Pel^SP^ Mutations on the Secretion Performance

The increased cutinase secretion of several of the obtained variants can be attributed to single amino acid substitutions within the Pel^SP^, as is for instance the case for V2, V4 and V7, which carry K3T, T8M or P16S, respectively (Table 2 and Figure 3). On the other hand, most isolated variants carry multiple DNA mutations within both the Pel^SP^ and the cutinase region (Table 2). In such cases, it is unclear what the contributions of the individual mutations are to the secretion performance. Moreover, in several variants silent mutations were identified. Although silent mutations do not alter the amino acid sequence of a protein, they can alter the mRNA folding/stability or influence the translation velocity, thus influencing the amount of protein that is produced (Brule and Grayhack, 2017) and subsequently secreted.

**FIGURE 4:**
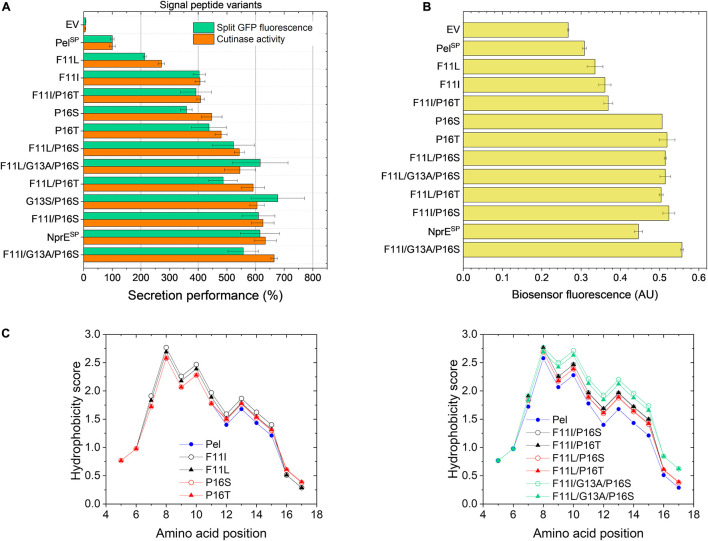
Secretion performance and hydrophobicity of Pel^SP^ variants created by site-directed mutagenesis. *C. glutamicum* K9 biosensor cells were transformed with pPBEx2-Pel-cutinase-GFP11 mutant plasmid encoding the different Pel^SP^ variants as indicated. The recombinant strains were grown in CGXII medium containing 1% (v/v) glucose in a BioLector for 24 h at 30°C, 1,200 rpm and 85% relative humidity, along with the control strains harboring pPBEx2 (EV), pPBEx2-NprE-cutinase-GFP11 (NprE^SP^) and pPBEx2-Pel-cutinase-GFP11 (Pel^SP^). Four hours after inoculation, IPTG was added to the cultures to a final concentration of 250 μM. **(A)** At the end of the cultivation (24 h), the secretion performance was assessed by measuring the split GFP fluorescence (green bars) and the cutinase activity (orange bars), respectively. The secretion performance of the different variants is shown relative to that of the Pel^SP^ control (100%) and is ranked according to the cutinase activity in ascending order. **(B)** The corresponding specific fluorescence at the end of the cultivation of the biosensor cells expressing the different variants is indicated. **(C)** Hydropathy plots showing the influence of the introduced single (left panel) and multiple mutations (right panel) as indicated on the overall hydrophobicity of the Pel^SP^. The data were obtained by ProtScale (https://web.expasy.org) and using the hydropathy scale according to Kyte and Doolittle (1982).

Based on the mutations found in the different variants (Table 2), potential beneficial mutations with a focus on the Pel^SP^ hotspot residues Phe11 and Pro16, were further evaluated. For this purpose, individual amino acid exchanges were introduced via site-directed mutagenesis at the designated positions into the WT Pel^SP^-cutinase-GFP11 gene. In addition, combinatorial mutants were created with the aim to improve secretion. First, the secretion performance of the Pel^SP^ single mutants F11I, F11L, P16S (V7) and P16T was investigated (Figure 4A). Notably, the sole introduction of the F11L mutation resulted in a ∼2-fold improved secretion performance, whereas the amino acid exchanges F11I, P16S, and P16T each resulted in ∼4-fold increase in secretion performance (Figure 4A). Interestingly, by combining these beneficial mutations, variants were obtained that showed a further improvement of secretion. In comparison to the Pel^SP^ control, the Pel^SP^ variants F11L/P16S, F11I/P16T and F11I/P16S showed a 5- to 6-fold increase in secretion performance. Moreover, the secretion performance of F11I/P16S equaled that of the NprE^SP^, which so far had proven to be a superior signal peptide for cutinase secretion in *C. glutamicum*. Thus, the replacement of only two amino acids was sufficient to convert the poor-performing Pel^SP^ into an excellent signal peptide for cutinase secretion. In an attempt to further improve secretion, two triple mutants were created. For this purpose, the G13A mutation as found in V7.1 (Table 2) was combined with the double mutations F11I/P16S and F11L/P16S. However, the corresponding triple mutants did however not show a significant further improvement of the secretion performance (Figure 4A).

The increase in the secretion performance as noted for the different Pel^SP^ variants is accompanied by an increase in the fluorescence output of the corresponding biosensor cells in all cases, indicating an increased secretion stress (Figure 4B). Although there is a general dose-dependent response of the specific fluorescence of the reporter strain with respect to the amounts (and activity) of the secreted cutinase, this correlation is not absolute. For instance, the biosensor fluorescence response was unable to resolve the secretion performance of the single mutants P16S/T and their derived multiple mutants that showed a better secretion performance (Figures 4A,B). It is important to note that the biosensor as such responds to the extent of stress associated with Sec-dependent protein export and not to the correctly folded, biologically active forms of the secreted target protein (Jurischka et al., 2020; Figure 1). In this respect, the introduction of the different mutations in the Pel^SP^ may have different effects on the secretion performance. The introduced mutation(s) may not only affect the number of cutinase molecules exported, by for instance altering the protein synthesis or the efficiency of export, but also the quality of the exported protein may be affected, e.g., by influencing the processing or the folding (before or after translocation). It can be envisaged that in cases where the folding is compromised, a larger proportion of incorrectly folded molecules will accumulate/aggregate in the periplasm, which triggers an increased secretion stress response (biosensor fluorescence). In cases where misfolding starts to compete with productive folding, less molecules are available for secretion, resulting in an increased biosensor fluorescence and a reduced secretion efficiency.

The hydrophobic properties of signal peptides are known to be particularly critical to the translocation process (Izard and Kendall, 1994; Freudl, 2018) and perturbations of the hydrophobic core by for instance the introduction of a charged or polar amino acid can decrease or even completely inhibit protein export (Chou and Kendall, 1990; Goldstein et al., 1991). Moreover, a minimal length and a minimum hydrophobic density of the h-region are essential for efficient protein translocation (Ryan et al., 1993; Wang et al., 2000; Duffy et al., 2010). In this regard, the Pel^SP^ (21 residues) is one of the shortest Sec signal peptides identified in *B. subtilis* (Brockmeier et al., 2006) and most of the amino acid mutations identified in the improved Pel^SP^ variants are located within the h-region (Table 2) and lead to an overall increase in the signal peptide hydrophobicity. The hydrophobicity profiles of the Pel^SP^ and several of its derived mutants are shown in Figure 4C.

The exchange of F11, which is at the center of the h-region, by the much more hydrophobic leucine or isoleucine results in a substantial increase in the overall hydrophobicity of the signal peptide (Figure 4C, left panel). As expected, the increase is highest for isoleucine, which is more hydrophobic. The increase in hydrophobicity of the signal peptide appears to be related to the secretion performance; showing a ∼2-fold and ∼4-fold improvement for F11L and F11I, respectively (*cf.* Figures 4A,C, left panel). Replacement of the hydrophilic Pro16 by the less hydrophilic serine or threonine also results in a small increase in signal peptide hydrophobicity (Figure 4C, left panel). In addition, the steric properties of proline also need consideration. Proline and glycine are helix breaker residues (Yaron and Naider, 1993) and substitution of Pro16 possibly eliminates a perturbation of the α-helical backbone conformation of the signal peptide, which in turn might alter the interaction of the signal peptide with (components of) the Sec-translocon. We speculate that the possible hydrophobic and steric effects associated with the P16S/T substitution contribute to the ∼4-fold improved secretion performance. Interestingly, for the *E. coli* outer-membrane protein PhoE it has been shown that removal of a helix breaker residue (i.e., Gly^–10^) in the h-region affected the targeting pathway, i.e., induced a switch from the post-translational (SecB-dependent) to a co-translational (SRP-dependent) export mode (Adams et al., 2002). At present, it remains unknown via which pathway(s) the cutinase is translocated across the cytoplasmic membrane of *C. glutamicum*.

In comparison to the signal peptide variants carrying the single mutations, their derived double mutants, as expected, all show a further increase in the overall hydrophobicity (Figure 4C, *cf*. left and right panel). For the double mutants F11I/P16S, F11L/P16S and F11L/P16T the increased hydrophobicity appears to correlate with a further increase in secretion performance, suggesting additive effects of the respective mutations. The double mutant F11I/P16T presents, however, an interesting exception. Combining the individual beneficial mutations F11I and P16T did not lead to an improvement of secretion (Figure 4A). In fact, the secretion performance of F11I/P16T was slightly lower than that of P16T, suggesting possible antagonistic effects of these combined mutations. The introduction of the G13A mutation in the well-secreted double mutants F11I/P16S and F11L/P16S also did not lead to a significant improvement of the secretion performance, even though the signal peptide hydrophobicity was strongly increased (Figure 4).

For improving recombinant protein secretion, there clearly is more to it than simply increasing the total hydrophobicity of the h-region of the signal peptide. On a protein level, the overall amino acid composition of the h-region, with different types of conformations and different degrees of side-chain hydrophobicity may contribute as well to efficient protein secretion (Izard and Kendall, 1994). Moreover, it can be envisaged that a too tight binding of the signal peptide to (one of) the Sec components might be disadvantageous in an export system that relies on a transient recognition by multiple interacting protein partners (Izard and Kendall, 1994). In addition, the various DNA mutations that were introduced for amino acid replacement can result in different mRNA transcripts that may have an altered structure and/or stability, which in turn may influence the amount of protein that is synthesized (Brule and Grayhack, 2017) and secreted.

### Secretion Performance of Selected Pel^SP^ Variants in Fed-Batch Cultivations

**FIGURE 5:**
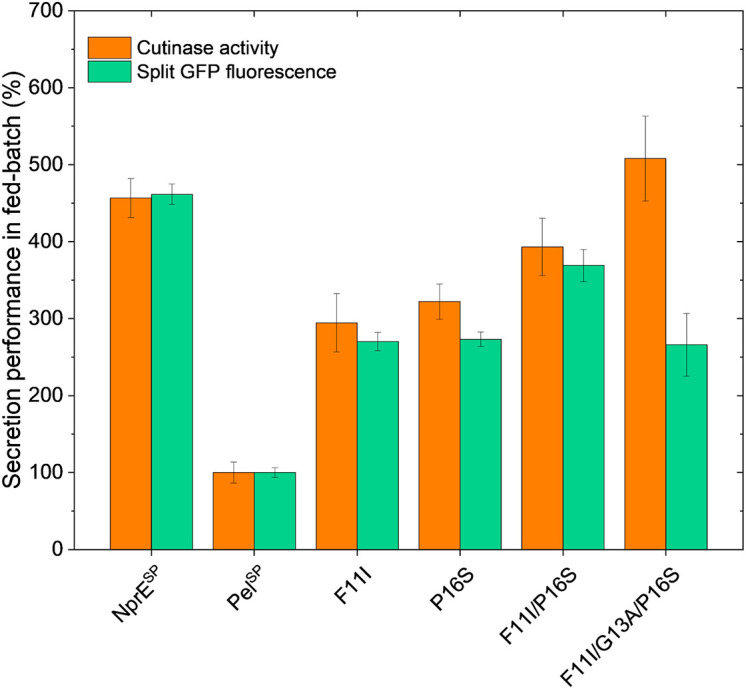
Fed-batch of *C. glutamicum* K9 biosensor cells harboring different recombinant plasmids for secretory production of cutinase-GFP11. Biosensor cells carrying pPBEx2-NprE-cutinase-GFP11 (NprE^SP^), pPBEx2-Pel-cutinase-GFP11 (Pel^SP^) or the Pel^SP^ variants F11I, P16S, F11I/P16S or F11I/G13A/P16S were cultivated in a BioLector Pro at 30°C, 1,400 rpm, ≥30% headspace oxygen and ≥85% relative humidity. For the fed-batch process, CGXII medium with an initial glucose concentration of 5 g/L was inoculated to an OD_600_ of 0.5 from the respective precultures. After 10 h, glucose was fed with a constant rate of 5.22 μL/h (equals 2.09 mg/h glucose). Regulation of pH to a set point of 6.8 was performed with 3 M KOH and was initiated 1 h after the start of the cultivation. Corresponding biomass and pH profiles are shown in Supplementary Figure S5. After 8 h of growth, cutinase-GFP11 expression was induced by the addition of IPTG (250 μM final concentration at the time of induction) and growth was continued for ∼17 h. At the end of the cultivation, the amount and activity of the secreted cutinase-GFP11 were determined by split GFP fluorescence and cutinase activity measurements, respectively.

While batch cultivation processes are predominantly used during the screening phase for novel strains or variants, fed-batch processes are state of the art for bioprocess development. However, batch conditions for screening can be error-prone, since variants with superior batch performance might underperform under fed-batch cultivation conditions in later stages (Hemmerich et al., 2019). Hence, fed-batch cultivations with selected Pel^SP^ variants were performed using micro-scale fed-batch cultivations. The obtained final data for the cutinase activity and the split GFP fluorescence measurements are shown in Figure 5. For comparability, the data were normalized with respect to the Pel^SP^ data. The standard error for the fed-batch data showed good reproducibility for all variants.

Importantly, the relative secretion performances of the different signal peptides under fed-batch conditions are in good agreement with those observed in batch cultivations (*cf.* Figures 4A, 5). Also in fed-batch, the NprE^SP^ clearly outperforms the Pel^SP^, showing a ∼450% higher secretion. In standard batch cultivations (250 μM IPTG/pPBEx2-based expression) the protein concentration of the extracellular cutinase-GFP11 achieved with the NprE signal peptide, as based on the split GFP fluorescence, was ∼0.5 mg/mL. For the more productive fed-batch cultivations, this would translate into a protein concentration of ∼1.5 mg/mL, which is amongst the highest concentrations reported for heterologous proteins secreted by *C. glutamicum*; i.e., endoxylanase XynA: 1.07 g/L, camelid antibody fragment (VHH): 1.57 g/L and Phospholipase C: 5.5 g/L (Ravasi et al., 2015; Yim et al., 2016).

Regarding the cutinase activities, the secretion ranking of the different signal peptides followed the order Pel^SP^ < F11I < P16S < F11I/P16S < NprE^SP^ < F11I/G13A/P16S. This confirms the observed additive effects of the Pel^SP^ mutations in the batch cultivations, although the performance of the F11I/P16S variant in batch cultivations was more similar to that of the NprE^SP^. Importantly, the highest activity of ∼13,000 U/L in fed-batch cultivations was achieved with the Pel^SP^ variant F11I/G13A/P16S. The cutinase activity for this variant was substantially higher than that achieved with the related F11I/P16S, whereas, interestingly, the corresponding split GFP fluorescence was substantially lower. A similar effect was noted in batch cultivations, albeit less pronounced (Figure 4A). Apparently, the cutinase secreted via the Pel^SP^ variant F11I/G13A/P16S possesses a higher specific activity (cutinase activity per split GFP fluorescence) than the cutinase secreted via variant F11I/P16S. Moreover, the Pel^L2^ variant V7.1, which carries the same G13A/P16S mutations (Table 2), also showed an increased specific activity (Figures 3A,C). These results may indicate that amino acid mutations in the signal peptide can affect the “quality” of the exported target protein, e.g., by influencing the signal peptide processing and/or the folding after translocation.

The successful transfer of the optimized signal peptide variants from batch to fed-batch cultivation demonstrates that the signal peptide screening approach with the protein secretion biosensor strain can deliver high performing strains not only under batch screening, but also under fed-batch conditions relevant for further development stages.

### Impact of the A85V Mutation on the Cutinase Secretion and Activity

**FIGURE 6:**
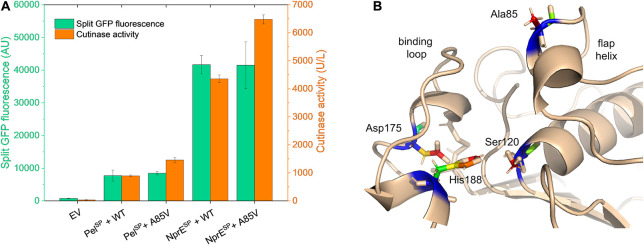
Influence of the mutation A85V on the cutinase secretion and activity. **(A)** *C. glutamicum* K9 biosensor cells were transformed with pPBEx2-Pel-cutinase-GFP11 or pPBEx2-NprE-cutinase-GFP11 plasmid harboring either wild-type (WT) or A85V cutinase, as indicated. Biosensor cells carrying pPBEx2 (EV) served as control. The recombinant strains were grown in CGXII medium containing 1% (v/v) glucose in a BioLector for 24 h at 30°C, 1,200 rpm and 85% relative humidity. Four hours after inoculation, IPTG was added to the cultures to a final concentration of 250 μM. At the end of the cultivation (24 h), the secretion performance was assessed by measuring the split GFP fluorescence (green bars) and the cutinase activity (orange bars), respectively. **(B)** 3D-representation of the X-ray structure of *Fusarium solani pisi* cutinase (PDB: 1CEX) showing the catalytic triad Ser120-Asp175-His188 and the binding loop, which are essential for cutinase activity. The cutinase mutation A85V, which increases the enzyme activity toward *p*NPP, maps to the flap helix, which lines up with the substrate-binding pocket.

Having successfully identified several mutations in the Pel^SP^ that enhance cutinase secretion, next, the impact of potential beneficial mutations in the mature cutinase part was investigated in more detail. For this purpose, we focused on the variant V3, which exhibits the highest specific activity (Figure 3C). V3 carries the F11L mutation in the signal peptide, as well as two silent mutations and the A85V exchange in the cutinase (Table 2). It is reasonable to assume that the increased specific activity of V3 is primarily due to the A85V mutation, but contributions of the F11L or the silent mutations cannot be excluded. Therefore, a cutinase variant harboring solely the A85V mutation was created. To facilitate secretion in batch cultivations, both the WT Pel^SP^ and NprE^SP^ were used. The secretion performance of the A85V variant is shown in comparison to that of the WT cutinase (Figure 6A). Notably, independent of the signal peptide used, the introduction of the A85V mutation did not significantly affect the amount of the secreted cutinase, whereas the activity of the mutant enzyme in the culture supernatant was substantially increased (Figure 6A). The observed increase in cutinase activity was ∼60% in case of Pel^SP^ and ∼50% in case of NprE^SP^, achieving ∼1,450 U/L and ∼6,400 U/L, respectively.

It is important to note that Ala85 lies within the substrate binding region of the enzyme, which is formed by the amino acids 40–52, 73–91, and 171–191 (Egmond and de Vlieg, 2000; Chen et al., 2013). Ala85 is part of the small flap helix (residues 81–85), which is located near the entrance of the substrate binding pocket (Figure 6B). Mutational analysis of cutinase from *F. solani pisi* has shown that replacement of Ala85 by a phenylalanine (or tryptophan) leads to an increase in enzyme activity on olive oil emulsions (Egmond and de Vlieg, 2000). A similar effect was observed for the cutinase CUTAB1 from *Alternaria brassicicola*. Here, replacement of the homologous Ala84 by a phenylalanine resulted in an increase in the activity of the enzyme towards longer chain substrates like *p*NPP (Koschorreck et al., 2010). It is suggested that the more hydrophobic phenylalanine in the helical flap increases the interactions with longer chain, hydrophobic substrates such as olive oil emulsions and *p*NPP (Egmond and de Vlieg, 2000; Koschorreck et al., 2010). We propose that the A85V mutation has a similar stimulatory effect on the enzyme activity of *F. solani pisi* cutinase. Although the A85V mutation did not alter the amount of the secreted cutinase, secretion of the A85V cutinase is accompanied by an increased secretion stress (Supplementary Figure S6). These results may indicate that the A85V mutation influences the folding of the enzyme after its translocation across the cytoplasmic membrane.

### Secretion Performance of Variants Obtained From an NprE^SP^-Cutinase-GFP11 Mutant Library

**FIGURE 7:**
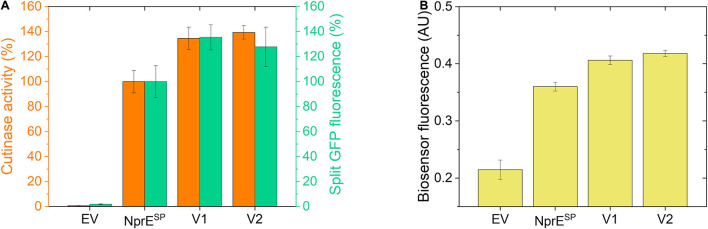
Secretion performance of two variants obtained from the NprE^SP^-cutinase-GFP11 mutant library. Random mutagenesis of pPBEx2-NprE-cutinase-GFP11 and subsequent FACS analysis and screening of biosensor cells expressing the mutant library was performed as described for the Pel^SP^-cutinase-GFP11 mutant libraries. Plasmids were isolated from promising clones and then re-introduced in the *C. glutamicum* K9 biosensor strain. The recombinant strains were grown in CGXII medium containing 1% (v/v) glucose in a BioLector for 24 h at 30°C, 1,200 rpm and 85% relative humidity along with the control strains harboring pPBEx2 (EV) or pPBEx2-NprE-cutinase-GFP11 (NprE^SP^). Four hours after inoculation, IPTG was added to the cultures to a final concentration of 250 μM. **(A)** At the end of the cultivation (24 h) the amount and activity of the extracellular cutinase-GFP11 was determined by split GFP fluorescence (green bars) and cutinase activity measurements (orange bars), respectively. **(B)** The specific fluorescence of the corresponding biosensor cells at the end of the cultivation (24 h) is indicated.

Finally, using the established biosensor-based approach, it was investigated whether the high-level cutinase secretion facilitated by the potent NprE^SP^ can be improved as well. First, an NprE^SP^ mutant library was created (in the same manner as described for Pel^SP^) that was subsequently expressed in the *C. glutamicum* K9 biosensor strain and then screened by FACS. Initial experiments revealed two promising clones that showed a substantial increase in the cutinase secretion. The plasmids were isolated from these original clones and then re-introduced in the K9 biosensor strain. The secretion performance of the resulting recombinant strains is shown in comparison to the NprE^SP^ control (Figure 7). Notably, the two obtained NprE^SP^ variants showed a significant increase in the secretion performance (Figure 7A), which is reflected by a substantial increase in biosensor fluorescence (Figure 7B). The secretion performance of the two variants was very similar; showing an approximate 35% increase in both the amount and activity of the extracellular cutinase (Figure 7A). DNA sequence analysis revealed that both variants carry the hydrophobic substitution S21I within the h-region of the signal peptide, whereas V2 in addition carries a silent mutation in the cutinase. These results highlight once more the importance of the signal peptide hydrophobicity in governing efficient protein secretion. The optimized variants NprE^SP^-S21I and Pel^SP^-F11I/G13A/P16S in comparison to their WT counterparts improved the secretory production of the cutinase from ∼4,000 to ∼5,500 U/L and from ∼700 to ∼4,100 U/L, respectively. These results may indicate that a well-performing signal peptide may have less room for improvement in comparison to a bad-performing signal peptide. Nevertheless, it was possible to substantially improve the secretion of the cutinase even when the excellent NprE^SP^ was used as a starting point for mutagenesis, which indicates that the maximum secretion capacity of *C. glutamicum* for cutinase had not been reached.

## Conclusion

In this study, we have successfully combined the previously developed K9 reporter strain (Jurischka et al., 2020) with the split GFP technology (Cabantous et al., 2005; Cabantous and Waldo, 2006; Knapp et al., 2017) to monitor cutinase secretion in *C. glutamicum* by means of fluorescence. Both fluorescence-based approaches allow the monitoring of Sec-dependent secretion of cutinase-GFP11 in a dose-dependent manner, independent of the usage of a direct activity assay for the target protein. The screening of the different mutant libraries using the fluorescent reporter strain in combination with high-throughput FACS proved to be a successful approach for the isolation of clones exhibiting an improved secretion performance, enabling the optimization of different signal peptides for cutinase secretion. The established biosensor-based approach is a particularly powerful tool for the optimization of an individual signal peptide for secretion, bypassing the need for the screening of large libraries of signal peptides from different Sec substrate proteins. Regarding the optimized signal peptides, it is difficult to determine the exact role of the identified mutations, as they can affect protein secretion on multiple different levels, including mRNA stability/folding, translation velocity, protein targeting and translocation as well as processing and folding. Nevertheless, many of the improved variants carry mutations in the h-region that increase the overall hydrophobicity of the signal peptide, indicating the general importance of the signal peptide hydrophobicity in governing efficient protein secretion. Therefore, targeted mutagenesis of the h-region would be a promising approach for further secretion improvement. Taken together, the biosensor-based approach for protein secretion optimization presented here offers great potential, as it can be applied to any desired signal peptide and target protein.

## Data Availability Statement

The original contributions presented in the study are included in the article/Supplementary Material, further inquiries can be directed to the corresponding author/s.

## Author Contributions

PB performed the conceptualization, investigation (implementation of split GFP assay, library construction, and screening), data analysis and visualization, and wrote the manuscript (original draft, review and editing). PL and AK contributed to the initial study design, provided plasmids and protocols for split GFP establishment, and contributed to the writing of the manuscript. CM performed the fed-batch experiments and contributed to the writing of the manuscript. AB constructed site-directed mutants and performed the cutinase activity measurements. DD-O performed the bacterial transformations and SDS-PAGE. MO, K-EJ, and RF contributed to the study design and the writing of the manuscript (original draft, review and editing). All authors have read and approved the manuscript.

## Conflict of Interest

AK is employed by Castrol Germany GmbH. The remaining authors declare that the research was conducted in the absence of any commercial or financial relationships that could be construed as a potential conflict of interest.

## Publisher’s Note

All claims expressed in this article are solely those of the authors and do not necessarily represent those of their affiliated organizations, or those of the publisher, the editors and the reviewers. Any product that may be evaluated in this article, or claim that may be made by its manufacturer, is not guaranteed or endorsed by the publisher.
