# Supplementary material for: Biosensor-Based Optimization of Cutinase Secretion by Corynebacterium glutamicum
Source: Front Microbiol. 2021 Oct 28;12:750150. doi: 10.3389/fmicb.2021.750150 (PMC8581548; doi:10.3389/fmicb.2021.750150)
Supplement: Supplementary file 1 [file Data_Sheet_1.pdf]

Supplementary Material

1 Supplementary Materials and Methods

Plasmid construction - The various plasmids and oligonucleotides required to create the expression constructs are shown in Supplementary Tables S1 and S2, respectively. For initial construction of pEKEx2-Pel-cutinase-GFP11 and pEKEx2-Pel-cutinase, plasmid pBSMul1-6nt-SPPel-Cut1-GFP11 was used as DNA template and PCR was performed to amplify the corresponding fusion gene with or without GFP11-tag using the respective primer pairs p01/p02 and p01/p03 (Supplementary Table S2). Primer p01 introduces the *C. glutamicum* consensus ribosome binding site AAGGAG (Supplementary Table S2). The corresponding PCR fragments were cleaved with *Pst*I and *Sac*I, and ligated into *Pst*I-*Sac*I treated pEKEx2. For construction of pEKEx2-NprE-cutinase-GFP11, PCR was performed on pBSMul1-6nt-SPPel-Cut1-GFP11, using primers p04 and p02 to amplify the C-terminal coding region of the cutinase-GFP11 gene. The resulting PCR fragment was cleaved with *Kpn*I and *Sac*I, and then ligated into *Kpn*I-*Sac*I-treated pEKEx2-NprE-cutinase. In all cases, after PCR, template DNA was eliminated by *Dpn*I treatment and the PCR products were purified. After ligation and transformation of *E. coli* DH5α with the various pEKEx2-derived constructs, recombinant plasmids were isolated and verified by DNA sequencing. The different cutinase fusion genes were subsequently transferred from the pEKEx2 plasmids as *Pst*I-*Sac*I fragments into the improved expression vector pPBEx2 (Bakkes et al., 2020).

For protein purification purposes, a His_6-tag was introduced in the NprE-cutinase-GFP11 fusion construct via oligonucleotide insertion. For this purpose, the complementary 5'-phosphorylated oligonucleotides p06 and p07 were used to generate double stranded DNA fragments encoding the His_6-tag with *Eco*RI-compatible overhangs. To this end, the primers p06 and p07 were mixed (10 µM each) in 10 mM Tris-HCl, pH 7.5 containing 50 mM NaCl and heated for 5 min at 95 °C in a thermomixer. Hybridization of the oligonucleotides was initiated by turning off the heater and allowing cool-down to room temperature. The hybridized fragment was then ligated with pPBEx2-NprE-cutinase-GFP11 which was *Eco*RI-cleaved and dephosphorylated beforehand.

In all cases, following ligation and transformation of *E. coli* with the different pPBEx2-derived constructs, recombinant plasmids were isolated and verified by DNA sequencing. Cloning of the initial expression constructs was performed using chemically competent *E. coli* DH5α (Table S1). Verified plasmids were electroporated into *C. glutamicum* biosensor cells as described previously (Eggeling and Bott, 2005). Transformants were grown on BHIS agar plates containing 25 µg/mL kanamycin, at 30°C for at least two days. Single clones were then picked and grown overnight in BHIS containing 25 µg/mL kanamycin, at 30°C and 250 rpm. Overnight cultures were supplemented with glycerol (20% v/v final concentration), flash-frozen using liquid nitrogen and then stored at -80°C until further use.

2 Supplementary Tables

Supplementary Table S1. Bacterial strains and plasmid used in this study.

**Bacterial strains**	**Properties**	**Source/reference**
E. coli DH5α	F^- Φ80*lac*ZΔM15 Δ(*lac*ZYA-*arg*F) U169 *rec*A1 *end*A1 *hsd*R17(r_k^-, m_k^+) *pho*A *sup*E44 *thi*-1 *gyr*A96 *rel*A1 λ^-	(Thermo Fisher Scientific, Langerwehe, Germany)
E. coli One Shot TOP10	*F- mcrA Δ(mrr-hsdRMS-mcrBC) φ80lacZΔM15 ΔlacX74 recA1 araD139 Δ(araleu)7697 galU galK rpsL (StrR) endA1 nupG*	(Invitrogen - Thermo Fisher Scientific, Langerwehe, Germany)
E. coli BL21(DE3)	*F– ompT gal dcm lon hsdSB(rB–mB–) λ(DE3 [lacI lacUV5-T7p07 ind1 sam7 nin5]) [malB+]K-12(λS)*	(Thermo Fisher Scientific, Langerwehe, Germany)
C. glutamicum ATCC13032 K9	*htrA*::*htrA´-eyfp* (replacement of *htrA* by *htrA´-eyfp*); biosensor strain used for secretion experiments.	(Jurischka et al., 2020)
**Plasmids**	**Properties**	**Source/reference**
pEKEx2	*E. coli*/*C. glutamicum* shuttle vector: $ori_{E.coli}$ from pUC18; $ori_{C.glu}$ from pBL1; P*tacI*; *lac*I^q; Kan^r.	(Eikmanns et al., 1994)
pEKEx2-NprE-cutinase	pEKEx2 containing a gene encoding the signal peptide from the *B. subtilis* protease NprE fused to the mature part of the cutinase from *F. solani pisi.* A *C. glutamicum* consensus *rbs* (AAGGAG) and adjoining spacer (ATATAGAT) are placed between the *Pst*I site and the ATG start codon of the fusion gene.	(Hemmerich et al., 2019;Bakkes et al., 2020)

pPBEx2	Cured pEKEx2-derivative containing a correct *lacI*^{q} allele and lacking destabilizing replicate sequences (Δ930-1140 nucleotides); Δ*Eco*RI site in the MCS; Kan^{r}.	(Bakkes et al., 2020)
pBSMul1(5nt)-Pel-cut11	pBSMul1 containing a HindIII–XbaI insert encoding a fusion protein of the signal peptide from *B. subtilis* pectate lyase (Pel) and *F. solani pisi* cutinase carrying a C-terminal GFP11-tag; Amp^{r}, Kan^{r}.	(Volkenborn et al., 2020)
pET22b-sfGFP1-10	Encodes detector fragment GFP 1-10 of a superfolder GFP variant (Cabantous et al., 2005) under control of a T7 promotor; Amp^{r}.	(Knapp et al., 2017)
pEKEx2-Pel-cutinase	pEKEx2 containing a *Pst*I–*Sac*I insert encoding a fusion protein of the signal peptide from *B. subtilis* pectate lyase (Pel) and cutinase from *F. solani pisi*.	This study
pEKEx2-Pel-cutinase-GFP11	pEKEx2 containing a *Pst*I–*Sac*I insert encoding a fusion protein of the Pel signal peptide and *F. solani pisi* cutinase carrying a C-terminal GFP11-tag.	This study
pEKEx2-NprE-cutinase-GFP11	pEKEx2 containing an insert encoding a fusion protein of the signal peptide from the *B. subtilis* neutral protease NprE and *F. solani pisi* cutinase carrying a C-terminal GFP11-tag.	This study
pPBEx2-Pel-cutinase	The insert from pEKEx2-Pel-cutinase was excised as a *Pst*I-*Sac*I fragment and ligated into pPBEx2.	This study

pPBEx2-Pel-cutinase-GFP11	The insert from pEKEx2-Pel-cutinase-GFP11 was excised as a *Pst*I-*Sac*I fragment and ligated into pPBEx2.	This study
pPBEx2-NprE-cutinase	The insert from pEKEx2-NprE-cutinase was excised as a *Pst*I-*Sac*I fragment and ligated into pPBEx2.	This study
pPBEx2-NprE-cutinase-GFP11	The insert from pEKEx2-NprE-cutinase-GFP11 was excised as a *Pst*I-*Sac*I fragment and ligated into pPBEx2.	This study
pPBEx2-NprE-H_6cutinase-GFP11	A His_6-encoding DNA fragment was inserted in frame between the NprE signal peptide and cutinase coding regions via the *Eco*RI restriction site.	This study
pPBEx2-Pel(F11I)-cuti11	Pel^{SP} mutant F11I fused to cutinase-GFP11.	This study
pPBEx2-Pel(F11L)-cuti11	Pel^{SP} mutant F11L fused to cutinase-GFP11.	This study
pPBEx2-Pel(P16S)-cuti11	Pel^{SP} mutant P16S fused to cutinase-GFP11 (V7 from Pel^{L1}).	This study
pPBEx2-Pel(P16T)-cuti11	Pel^{SP} mutant P16T fused to cutinase-GFP11.	This study
pPBEx2-Pel(F11I/P16S)-cuti11	Pel^{SP} mutant F11I/P16S fused to cutinase-GFP11.	This study
pPBEx2-Pel(F11I/P16T)-cuti11	Pel^{SP} mutant F11I/P16T fused to cutinase-GFP11.	This study
pPBEx2-Pel(F11L/P16S)-cuti11	Pel^{SP} mutant F11L/P16S fused to cutinase-GFP11.	This study
pPBEx2-Pel(F11L/P16T)-cuti11	Pel^{SP} mutant F11L/P16T fused to cutinase-GFP11.	This study

pPBEx2-Pel(F11I/G13A/P16S)-cuti11	Pel^{SP} mutant F11I/G13A/P16S fused to cutinase-GFP11.	This study
pPBEx2-Pel(F11L/G13A/P16S)-cuti11	Pel^{SP} mutant F11L/G13A/P16S fused to cutinase-GFP11.	This study
pPBEx2-Pel-cuti11(A85V)	Wild-type Pel^{SP} fused to cutinase-GFP11 mutant A85V.	This study
pPBEx2-NprE-cuti11(A85V)	Wild-type $NprE^{SP}$ fused to cutinase-GFP11 mutant A85V.	This study

Supplementary Table S2. Oligonucleotides used to create the various cutinase fusion genes.

Primer name	Sequence (5’→3’)
**Oligonucleotides for fusion gene construction and error prone PCR**	
p01_PstI-Pel-f	CATGCCTGCAGAAGGAGATATAGATATGAAAAAGTGATGTTAGCTACGGCTTTGT
p02_SacI-GFP11-r	TGACTTGCATGAGCTCTTATGTGATGCCAGCAGCGTTAACGTATTCA
p03_SacI-Cut-r	TGACTTGCATGAGCTCTTAAGCAGAACCACGGACAGCCCGAACCT
p04_KpnI-Cut-f	CCTCGCGGTACCTCTAGCGCCGCAATC
p05_His6-f	[PHO]-AATTCGCGCACCATCACCACCACCATGCAG
p06_His6-r	[PHO]-AATTCTGCATGGTGGTGGTGATGGTGCGCG
p07_ep_PstI-f	GCTTGCATGCCTGCAGAAGGAGATATAGAT
p08_ep_KpnI-r	TGATTGCGGCGCTAGAGGTACCG
**Oligonucleotides for site-directed mutagenesis**	
p09_P16S-f	TTGACT**T**CAGCTGGCGCGAACGCAGCTG
p10_P16T-f	TTGACT**A**CAGCTGGCGCGAACGCAGCTG
p11_P16-r	TCCTAAAAACAAAGCCGTAGCTAACATC
p12_F11-f	TTGACTCCAGCTGGCGCGAACGCAGCTG
p13_F11I-r	TCCTAAAA**T**CAAAGCCGTAGCTAACATC

p14_F11L-r	TCCTAA**<u>T</u>**AACAAAGCCGTAGCTAACATC
p15_F11I/G13A-r	T**<u>G</u>**CTAAAA**<u>T</u>**CAAAGCCGTAGCTAACATC
p16_F11L/G13A-r	T**<u>G</u>**CTAA**<u>T</u>**AACAAAGCCGTAGCTAACATC
p17_cuA85V-f	ACAATG**<u>T</u>**TCTCCCTCGCGGTACCTCTAGC
p18_cuA85V-r	CTCCAAGAGTGGCTCGGTAGGCACCG

Relevant restriction sites used for cloning are underlined; desired point mutations for site-directed mutagenesis are in bold and underlined.

Supplementary Table S3. Variants created by site-directed mutagenesis**.**

Construct short name	Forward primer	Reverse primer	DNA template
**Signal peptide variants**			
P16S			Pel-cutinase-GFP11 plasmid from clone V7 isolated from Pel^{L1} harbors the P16S mutation in the Pel^{SP}
P16T	p10	p11	Pel-cutinase-GFP11
F11I	p12	p13	Pel-cutinase-GFP11
F11L	p12	p14	Pel-cutinase-GFP11
F11I/P16S	p09	p13	Pel-cutinase-GFP11
F11L/P16S	p09	p14	Pel-cutinase-GFP11
F11I/P16T	p13	p10	P16T
F11L/P16T	p14	p10	Pel-cutinase-GFP11
F11I/G13A/P16S	p09	p15	F11I/P16S
F11L/G13A/P16S	p09	p16	F11L/P16S
**Cutinase variants**			
Pel-cuA85V	p17	p18	Pel-cutinase-GFP11
NprE-cuA85V	p17	p18	NprE-cutinase-GFP11

Supplementary Table S4. Overview of the FACS sorting conditions and assessment of the selectivity for cutinase producing clones isolated from the Pel^{L1} mutant library.

Enrichment conditions [a]				Single cell sort [b]	Cutinase producing clones [c]		
	Sort gate	Pel^{L1} cells	IPTG (µM)	Sort gate	-	+	+ (%)
A	P3-M	200.000	250	P3-1	79	9	10.2
	P3-M	100.000	250	P3-1	80	8	10.0
B	P3-1	200.000	250	P3-1	60	28	31.8
	P3-1	200.000	250	P3-2	59	29	33.0
	P3-1	200.000	1000	P3-2	70	18	20.4
C	P3-2	100.000	250	P3-1	55	33	37.5
	P3-2	100.000	250	P3-2	50	38	43.2
	P3-2	100.000	1000	P3-2	64	24	27.3

[a] Pel^{L1} cells were sorted into CGXII medium containing 1% (v/v) glucose and IPTG at indicated final concentration. Cells were subsequently cultivated in a BioLector system (enrichment step).

[b] Individual Pel^{L1} cells were sorted from the enriched cultures onto BHIS-agar plates containing 15 µg/ml kanamycin and grown at 30°C for at least two days.

[c] The cutinase production phenotype of the individual clones was assessed by an *in situ* biological assay on Tween-agar plates (Bakkes et al., 2020).

3 Supplementary Figures

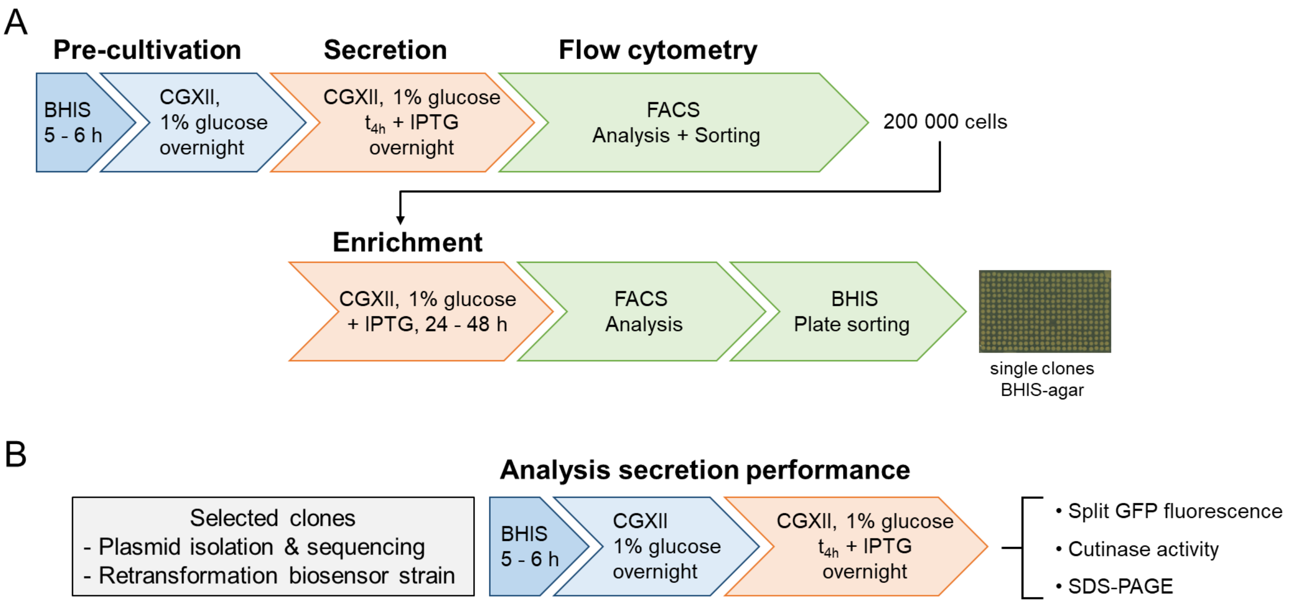

Supplementary Figure S1. Biosensor-based screening of a mutant library for variants exhibiting improved cutinase-GFP11 secretion. (A) The *C. glutamicum* biosensor cells harbouring a mutant library were grown in BHIS medium for 5 – 6 h and then transferred to CGXII medium containing 1% (v/v) glucose in a FlowerPlate. The cells were then cultivated in a BioLector system at 30°C, 1200 rpm and 85% relative humidity. Biosensor cells carrying pPBEx2 (empty vector), pPBEx2-Pel-cutinase-GFP11 or pPBEx2-NprE-cutinase-GFP11 served as controls. The overnight cultures were used to inoculate fresh CGXII medium containing 1% (v/v) glucose, using a starting OD600 of 1. After 4 hours of growth (30°C, 1200 rpm and 85% relative humidity), IPTG was added (usually 250 µM final concentration) to induce the expression of the respective cutinase genes. The next day, the cultures were analysed by FACS and highly fluorescent cells (typically 200 000) were sorted directly into CGXII medium containing 1% (v/v) glucose and IPTG (typically 250 µM). The cells were grown in the BioLector system until the stationary phase was reached (enrichment cultivation). The cultures were then analysed by FACS and single cells were sorted onto BHIS-agar plates (OmniTray) containing 15 µg/ml kanamycin. The plates were incubated at 30°C for at least 2 days to allow for the development of colonies. Typically, the sorting efficiency was >97%. Hereafter, the secretion performance of the sorted clones was assessed by a method of choice, e.g. the pNPP assay or Tween-agar plates for assessing cutinase activity, or the split GFP assay for activity-independent screening. (B) To confirm that the improved cutinase secretion is plasmid-linked, plasmid DNA was isolated from the original clones and then re-introduced in the biosensor strain. In all cases, at least two independent clones were analysed in a standard BioLector secretion experiment. The secretion performance was assessed by determining the amount (split GFP assay and/or SDS-PAGE analysis) and the activity (pNPP assay) of the extracellular cutinase. The split GFP fluorescence and cutinase activity measurements were carried out in duplicate.

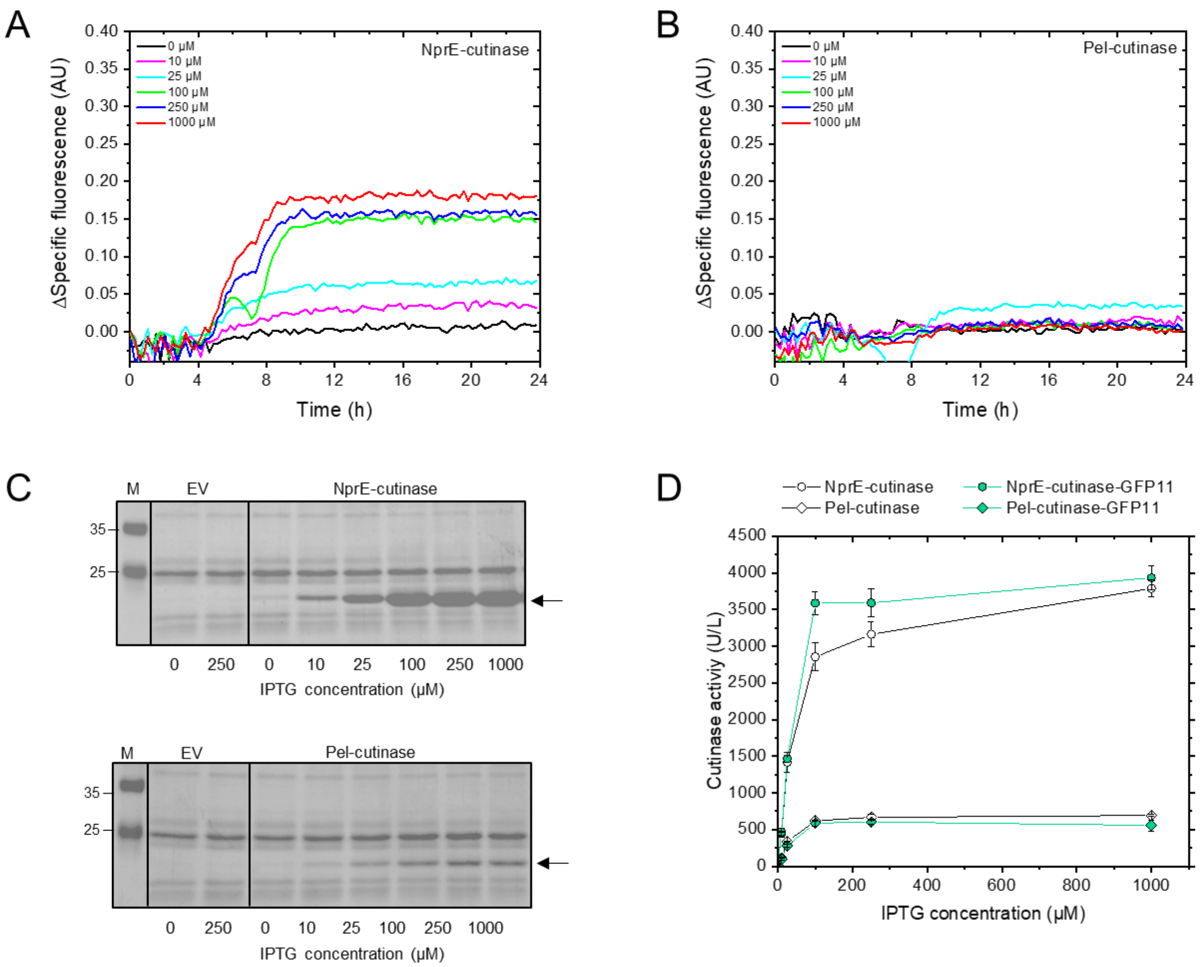

Supplementary Figure S2. Monitoring the secretion of cutinase by the *C. glutamicum* fluorescent (eYFP) K9 biosensor strain and split GFP technology. *C. glutamicum* K9 biosensor cells harbouring pPBEx2 (EV), pPBEx2-NprE-cutinase or pPBEx2-Pel-cutinase were grown in CGXII medium in a 48-well FlowerPlate in a BioLector system for 24 h at 30°C, 1200 rpm and 85% relative humidity. Four hours after inoculation (start OD_{600} ~1), IPTG was added to the cultures to indicated final concentrations. The fluorescence response of the biosensor cells during cultivation is shown as the Δspecific fluorescence over time for cells expressing (A) NprE-cutinase or (B) Pel-cutinase (B). (C) Cutinase in the supernatants of the respective cultures was analysed after 24 h of growth by SDS-PAGE. The proteins were visualised by Coomassie Brilliant Blue staining. EV; *C. glutamicum* K9 biosensor cells harbouring pPBEx2 empty vector. The arrows indicate the position of the processed (signal peptide-less) cutinase (expected size 22.1 kDa) (D) The amount and activity of the cutinase in the culture supernatants was in addition determined via split GFP and pNPP measurements, respectively.

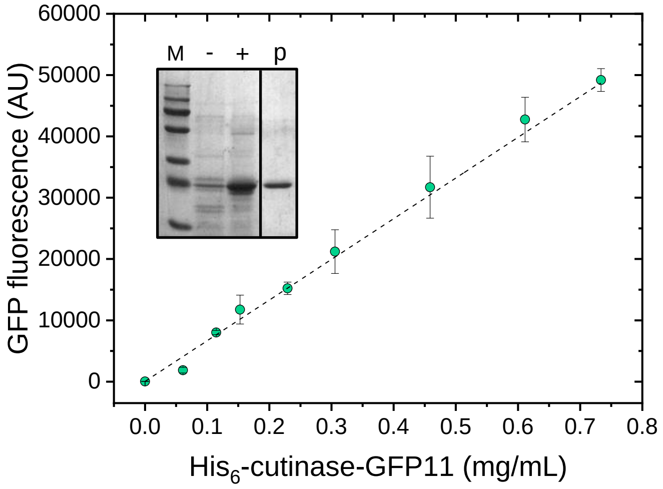

Supplementary Figure S3. Correlation between the amounts of GFP11-tagged cutinase and the GFP fluorescence after reconstitution of the holo-GFP. To facilitate purification of His_6-cutinase-GFP11 from the culture medium via immobilised metal ion affinity chromatography (IMAC), a His_6-tag was introduced between the $NprE^{SP}$ and cutinase-GFP11. The His-tagged cutinase-GFP11 is effectively secreted. The inset shows the SDS-PAGE analysis of the culture supernatants of non-induced (-) and induced (+, 250 µM IPTG) K9 biosensor cells carrying pPBEx2-NprE-H6cutinase-GFP11 (after 24 h of growth). Notably, secretion levels were similar to those obtained for cutinase-GFP11 lacking the N-terminal His_6-tag. IMAC-purified His_6-cutinase-GFP11 is shown in the right lane (p). A standard split GFP assay was then performed using different amounts of the purified His_6-cutinase-GFP11 in the presence of excess detector GFP1-10. The results indicate a good linear relation between the applied amount of protein and the developed fluorescence (y = 66358.9x + 35.6; R^2 = 0.9985). The use of known amounts of His_6-cutinase-GFP11 thus allows generation of a calibration curve, which in turn can be used to quantify the amounts of extracellular cutinase-GFP11 in secretion cultures (mg/mL), solely based on the reconstituted GFP fluorescence. Under standard batch conditions in BioLector cultivations (250µM IPTG / pPBEx-based expression) the extracellular cutinase-GFP11 secreted via the NprE signal peptide has a protein concentration of ~0.5 mg/mL.

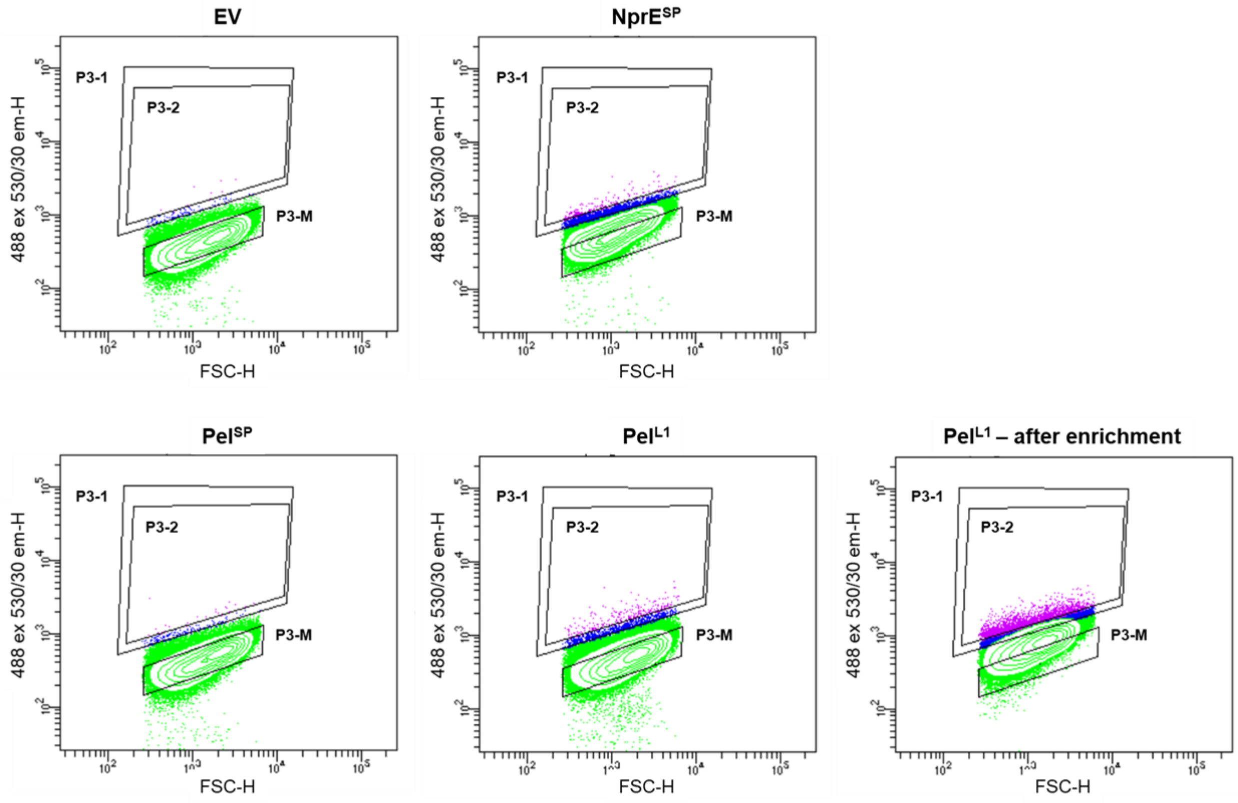

Supplementary Figure S4. FACS analysis of *C. glutamicum* K9 biosensor cells harbouring different recombinant plasmids for secretory production of cutinase-GFP11. A standard secretion experiment was carried out in a BioLector system using *C. glutamicum* biosensor cells harbouring pPBEx2 (EV), pPBEx2-NprE-cutinase-GFP11 (NprE^{SP}), pPBEx2-Pel-cutinase-GFP11 (Pel^{SP}) or the mutant library of pPBEx2-Pel-cutinase-GFP11 (Pel^{L1}). In all cases, induction was carried out using IPTG at a final concentration of 250 µM IPTG. At the end of the cultivation (24 h), cells were subjected to FACS analysis as described in the Materials and Methods section in the main manuscript. For all FACS experiments, a preselection of cells was performed to exclude cell doublets and cell debris by gating in a dot plot of FSC-H against FSC-W (gate P2). The dot plots show analysis of $1.0 \cdot 10^5$ P2 cells for which the eYFP fluorescence (y-axis) is plotted against the cell size (FSC-H). The different applied sorting gates, P3-M, P3-1 and P3-2 are indicated. Using gate P3-1, $2.0 \cdot 10^5$ cells were sorted from Pel^{L1} directly into CGXII medium containing 1% (v/v) glucose and 250 µM IPTG and then cultivated in BioLector system until the stationary phase was reached. Analysis of the corresponding Pel^{L1} cells after enrichment cultivation is shown in the lower right graph.

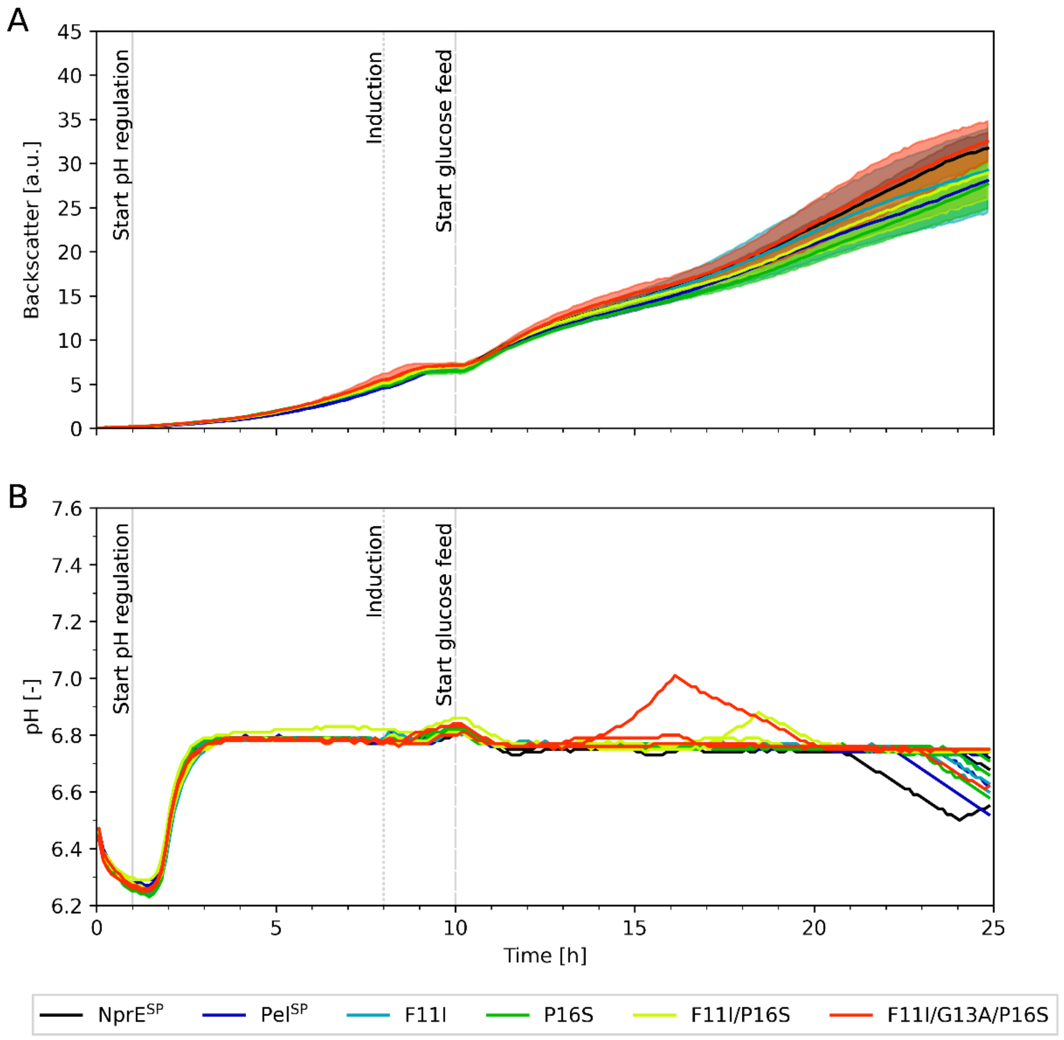

Supplementary Figure S5. Fed-batch of *C. glutamicum* K9 biosensor cells harbouring different recombinant plasmids for secretory production of cutinase-GFP11. Biosensor cells carrying pPBEx2-NprE-cutinase-GFP11 (NprE^{SP}), pPBEx2-Pel-cutinase-GFP11 (Pel^{SP}) or the Pel^{SP} variants F11I, P16S, F11I/P16S or F11I/G13A/P16S were cultivated in a BioLector Pro at 30°C, 1400 rpm, ≥30% headspace oxygen and ≥85% relative humidity. Biomass (A) and pH (B) were measured online with error bars in biomass measurement originating from ≥3 replicates. CGXII medium with an initial glucose concentration of 5 g/L was inoculated to an OD_{600} of 0.5 from the respective precultures. After 10 h, glucose was fed with a constant rate of 5.22 µL/h (equals 2.09 mg/h glucose). Regulation of pH to a set point of 6.8 was performed with 3 M KOH and was initiated after 1 h. A drop in the pH value at the beginning of the experiment is due to the interference of the optical measurement by the protocatechuic acid in the medium (Morschett et al., 2020). Cutinase-GFP11 expression was induced using 250 µM IPTG (final concentration at the time of induction) after 8 h. The supernatants were taken after ~25 h and analyzed with respect to the cutinase amount and activity by means of the split GFP and pNPP assay, respectively.

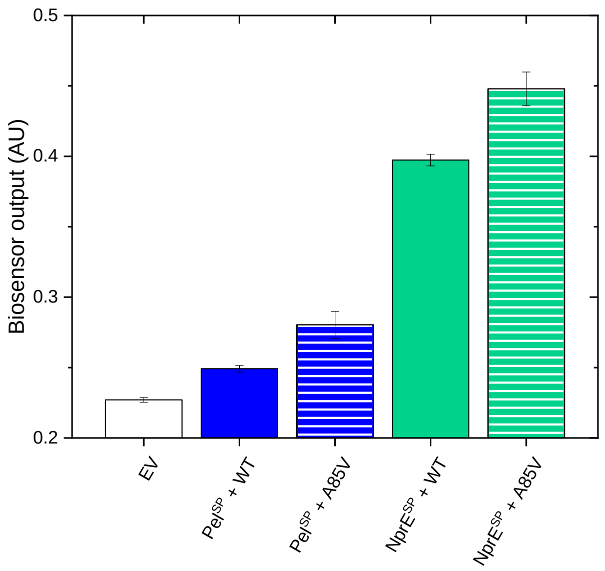

Supplementary Figure S6. Fluorescence output of *C. glutamicum* K9 biosensor cells secreting cutinase-GFP11 that carries the helical flap mutation A85V. *C. glutamicum* K9 biosensor cells were transformed with pPBEx2-Pel-cutinase-GFP11 or pPBEx2-NprE-cutinase-GFP11 plasmid encoding either the wild-type or A85V cutinase. Biosensor cells harbouring pPBEx2 (EV) served as control. The recombinant strains were grown in CGXII medium containing 1% (v/v) glucose in a BioLector system for 24 h at 30°C, 1200 rpm and 85% relative humidity. Four hours after inoculation, IPTG was added to the cultures to a final concentration of 250 µM. The biosensor output, i.e. the specific eYFP fluorescence of the respective biosensor cells at the end of the cultivation (24 h) is shown.

4 References

Bakkes, P.J., Ramp, P., Bida, A., Dohmen-Olma, D., Bott, M., and Freudl, R. (2020). Improved pEKEx2-derived expression vectors for tightly controlled production of recombinant proteins in *Corynebacterium glutamicum*. *Plasmid* 112**,** 102540. DOI: 10.1016/j.plasmid.2020.102540.

Cabantous, S., Terwilliger, T.C., and Waldo, G.S. (2005). Protein tagging and detection with engineered self-assembling fragments of green fluorescent protein. *Nat Biotechnol* 23**,** 102-107. DOI: 10.1038/nbt1044.

Eggeling, L., and Bott, M. (2005). *Handbook of Corynebacterium glutamicum.* Boca Raton: CRC Press. DOI: 10.1201/9781420039696

Eikmanns, B.J., Thum-Schmitz, N., Eggeling, L., Ludtke, K.U., and Sahm, H. (1994). Nucleotide sequence, expression and transcriptional analysis of the *Corynebacterium glutamicum gltA* gene encoding citrate synthase. *Microbiology* 140 (Pt 8)**,** 1817-1828. DOI: 10.1099/13500872-140-8-1817.

Hemmerich, J., Moch, M., Jurischka, S., Wiechert, W., Freudl, R., and Oldiges, M. (2019). Combinatorial impact of Sec signal peptides from *Bacillus subtilis* and bioprocess conditions on heterologous cutinase secretion by *Corynebacterium glutamicum*. *Biotechnol Bioeng* 116**,** 644-655. DOI: 10.1002/bit.26873.

Jurischka, S., Bida, A., Dohmen-Olma, D., Kleine, B., Potzkei, J., Binder, S., et al. (2020). A secretion biosensor for monitoring Sec-dependent protein export in *Corynebacterium glutamicum*. *Microb Cell Fact* 19**,** 11. DOI: 10.1186/s12934-019-1273-z.

Knapp, A., Ripphahn, M., Volkenborn, K., Skoczinski, P., and Jaeger, K.E. (2017). Activity-independent screening of secreted proteins using split GFP. *J Biotechnol* 258**,** 110-116. DOI: 10.1016/j.jbiotec.2017.05.024.

Morschett, H., Jansen, R., Neuendorf, C., Moch, M., Wiechert, W., and Oldiges, M. (2020). Parallelized microscale fed-batch cultivation in online-monitored microtiter plates: implications of media composition and feed strategies for process design and performance. *J Ind Microbiol Biotechnol* 47**,** 35-47. DOI: 10.1007/s10295-019-02243-w.

Volkenborn, K., Kuschmierz, L., Benz, N., Lenz, P., Knapp, A., and Jaeger, K.E. (2020). The length of ribosomal binding site spacer sequence controls the production yield for intracellular and secreted proteins by *Bacillus subtilis*. *Microb Cell Fact* 19**,** 154. DOI: 10.1186/s12934-020-01404-2.
